# Frailty associates with respiratory exacerbations and mortality in the COPDGene cohort

**DOI:** 10.18632/aging.206275

**Published:** 2025-07-03

**Authors:** Eleanor Kate Phillips, Yichen Huang, Elizabeth Regan, Barry Make, Matthew Strand, Abebaw Mengistu Yohannes, Nicola A. Hanania, Jessica Bon, Karin F. Hoth, James D. Crapo, Edwin K. Silverman, Dawn L. DeMeo

**Affiliations:** 1 Channing Division of Network Medicine, Brigham and Women's Hospital, Boston, MA 02115, USA; 2 Division of Pulmonary and Critical Care Medicine, Brigham and Women's Hospital, Boston, MA 02115, USA; 3 Division of Rheumatology, National Jewish Health, Denver, CO 80206, USA; 4 Division of Pulmonary, Critical Care, and Sleep Medicine, National Jewish Health, Denver, CO 80206, USA; 5 Division of Biostatistics and Bioinformatics, National Jewish Health, Denver, CO 80206, USA; 6 Department of Physical Therapy, University of Alabama at Birmingham, Birmingham, AL 35294, USA; 7 Section of Pulmonary and Critical Care Medicine, Baylor College of Medicine, Houston, TX 77030, USA; 8 Section of Pulmonary, Critical Care, Allergy, and Immunologic Diseases, Wake Forest University School of Medicine, Winston-Salem, NC 27101, USA; 9 Department of Psychiatry, University of Iowa Carver College of Medicine, Lowa City, IA 52242, USA; 10 Harvard Medical School, Boston, MA 02115, USA

**Keywords:** frailty, cigarette smoking, respiratory exacerbations, COPD, epigenetic aging

## Abstract

Frailty is associated with respiratory exacerbations and mortality in individuals with Chronic Obstructive Pulmonary Disease (COPD). Among those with a smoking history and normal spirometry, frailty’s association with respiratory outcomes is less defined.

COPDGene is a cohort study of individuals aged 45–80 with a minimum 10 pack-year smoking history. A modified Fried Frailty Phenotype was performed at 10-year follow-up; participants were categorized as frail, prefrail, or robust. Primary outcomes were respiratory exacerbations, epigenetic pace of aging, and all-cause mortality.

Among 2665 participants, 401 (15%) were frail and 1352 (51%) were prefrail. Adjusting for smoking and lung function, frailty was associated with prospective respiratory exacerbation rate (IRR 3.4, 95% CI 2.4–4.8), severe exacerbations (OR 2.8(1.8–4.2)), and frequent exacerbations (OR 5.5(3.2–9.3)). Prefrailty was also associated with exacerbation outcomes (rate IRR 1.8(1.4–2.3); severe OR 1.6(1.1–2.2); frequent OR 2.6(1.7–4.1)). Frailty and prefrailty were associated with increased all-cause mortality (AHR: frailty 4.5(2.4–8.5); prefrailty 2.5(1.5–4.2)). All frailty (and most prefrailty) findings persisted in those with normal spirometry. Baseline DunedinPACE of aging was associated with prospective frailty at 10-year follow-up.

Frailty associated with respiratory exacerbations and mortality; findings persisted among individuals with normal spirometry, highlighting the relevance of evaluating for frailty in people with a history of smoking.

## INTRODUCTION

Frailty is a syndrome of decreased functional reserve and increased vulnerability to stressors. It has been associated with advanced age, chronic diseases including chronic obstructive pulmonary disease (COPD), and increased risk of disability and death [1]. A commonly used method for assessing frailty is the Fried Frailty Phenotype (FFP), a physiologic definition that categorizes individuals as frail, prefrail, or robust based on five components: shrinking, weakness, slowness, low activity level, and fatigue [1].

The reported prevalence of frailty in populations with COPD varies from 6–58% [2–10]. COPD is associated with increased odds of frailty and with increased rates of frailty progression [2, 11]. Among individuals with COPD, frailty increases the risk of hospitalizations and death [2, 5, 7]. Frailty (as well as handgrip weakness, a component of the frailty definition) has been associated with increased risk of COPD exacerbations [5, 12–15], although this association has not been consistently demonstrated [16]. Notably, in individuals with COPD, completing a pulmonary rehabilitation program may reverse the frailty phenotype [17].

Prefrailty, a potential ‘subclinical’ precursor to frailty, has also been linked to adverse outcomes [1, 18]. Among individuals with COPD, prefrailty has been associated with respiratory exacerbations as defined by electronic medical record codes and drug prescription data [5]. Its association with mortality in this population has been less consistent, although a recent meta-analysis demonstrated a pooled hazard ratio of 1.5 (0.9–2.4) of prefrailty on all-cause mortality [5, 19].

While cigarette smoking has been associated with prevalent frailty [20–22], the association between smoking and frailty development has been variable [23–28]. In particular, one study found that current smoking was associated with two-fold odds of incident frailty, but this effect was not observed after adjusting for COPD status [29]. The associations between smoking and frailty in the literature have not been consistently adjusted for lung function or COPD status, potentially contributing to this variability of findings.

The burden of smoking-related symptoms among individuals with normal spirometry is becoming increasingly recognized [30, 31]. Compared to never-smokers, populations with a cigarette smoking history and normal spirometry have demonstrated more respiratory exacerbations, higher dyspnea scores, higher airway wall thickness, and more evidence of radiographic emphysema [30, 31]. The relationship between frailty and respiratory exacerbations in this population remains unclear. While Verschoor and colleagues identified a cross-sectional association between history of respiratory symptoms (any cough, wheeze, or dyspnea in the past year) and frailty [32], this has not to our knowledge been studied prospectively, nor with a focus on exacerbations and with consideration of prefrailty.

Prior studies have demonstrated associations between a variety of epigenetic age acceleration measures and frailty [33–35]. DunedinPACE, a novel DNA methylation-based biomarker of the pace of aging, has been associated with subsequent (7-year) frailty in a small study of older adults (aged ≥70) [36, 37]. Another recent study suggested that a higher DunedinPACE may predate changes in frailty [38]. Given the extensive impact of current cigarette smoking on the epigenome, and noting that associations between smoking-related DNA methylation changes and frailty have been observed, we performed a smoking-stratified assessment of the association between DunedinPACE and frailty at 10-year follow up [21].

In this study, we determined the prevalence of frailty and prefrailty in a population with a smoking history and evaluated their associations with subsequent respiratory exacerbations and all-cause mortality. To elucidate the associations between frailty and outcomes independent of COPD, we adjusted for lung volume in regression models. We also performed subgroup analyses of individuals with normal spirometry, mild COPD (Global Initiative for Chronic Obstructive Lung Disease (GOLD) 1), moderate to very severe COPD (GOLD 2–4), and Preserved Ratio Impaired Spirometry (PRISm). We additionally conducted post-hoc analyses comparing smoking intensity to frailty and evaluating frailty in a cohort of never-smoker controls. We hypothesized that frailty and prefrailty would increase the risk of adverse outcomes across spirometric subgroups, including among those with normal spirometry.

## RESULTS

### Demographics and baseline characteristics

Of 2665 participants, 401 (15%) were frail, and 1352 (51%) were pre-frail (Table 1). The mean age (standard deviation) of the study population was 70(8). The distribution of frailty category by age was similar for subjects between 50–80 years old; frailty prevalence was increased among individuals aged 80 and above (Supplementary Figure 1).

**Table 1:** Participant characteristics.

**Characteristic**	***n***	**Robust**	**Prefrail**	**Frail**	*p* ^*^
*n* (%)	2,665	912 (34%)	1,352 (51%)	401 (15%)	
Age	2,665	68.6 (7.4)	69.7 (8.1)	71.5 (9.2)	**<0.001**
Sex	2,665				0.39
Male		450 (49.3%)	640 (47.3%)	204 (50.9%)	
Female		462 (50.7%)	712 (52.7%)	197 (49.1%)	
Race	2,665				**<0.001**
Non-Hispanic White		742 (81.4%)	933 (69.0%)	270 (67.3%)	
African American		170 (18.6%)	419 (31.0%)	131 (32.7%)	
BMI	2,665	28.6 (5.5)	28.5 (6.1)	30.1 (7.6)	**0.002**
Current Smoking	2,663	239 (26.2%)	462 (34.2%)	138 (34.4%)	**<0.001**
Smoking Pack-Years	2,663	39.3 (20.3)	43.0 (22.4)	52.7 (26.6)	**<0.001**
GOLD grade	2,646				**<0.001**
Normal Spirometry		473 (52.3%)	589 (43.7%)	108 (27.4%)	
1		115 (12.7%)	154 (11.4%)	27 (6.9%)	
2		160 (17.7%)	293 (21.8%)	68 (17.3%)	
3		56 (6.2%)	104 (7.7%)	85 (21.6%)	
4		9 (1.0%)	32 (2.4%)	52 (13.7%)	
Total GOLD 2–4 (Moderate-Severe COPD)		225 (24.9%)	429 (31.8%)	205 (52.0%)	**<0.001**
PRISm		92 (10.2%)	175 (13.0%)	54 (13.7%)	0.077
Comorbidity Count	2,665	1.1 (1.1)	1.4 (1.2)	2.0 (1.4)	**<0.001**

Total *N* = 2665. *N* with data available for each characteristic shown. Continuous variables reported as mean (standard deviation). Categorical variables reported as *n* (%). Abbreviations: BMI: body mass index (kg/m^2^). GOLD: Global Initiative for Chronic Obstructive Lung Disease; COPD: Chronic Obstructive Pulmonary Disease; PRISm: Preserved Ratio Impaired Spirometry. Comorbidity count is the sum of the following reported comorbidities: diabetes, coronary artery disease (including reported coronary artery disease, myocardial infarction, angina, angioplasty, or coronary artery bypass graft surgery), congestive heart failure, cerebrovascular disease (including reported stroke or transient ischemic attack), kidney disease, liver disease, cancer (excluding non-melanoma skin cancer), osteoarthritis, and osteoporosis. Expanded characteristics are available in Supplementary Table 1. ^*^p-values are calculated by the Kruskal-Wallis rank sum test for continuous variables and by Pearson’s chi-squared test for categorical variables.

Frailty prevalence was higher among individuals with GOLD 2–4 COPD (24%) than in those with normal spirometry (9%) (Supplementary Figure 1). Comorbidities including cardiovascular disease and osteoarthritis were associated with frailty. Frailty distribution across BMI categories was U-shaped (Supplementary Figure 1). Frail subjects were more likely to report needing assistance with basic and independent activities of daily living (BADLs/IADLs); 25% of frail subjects reported needing help with IADLs, compared to less than 1% of robust subjects. Frailty category was also associated with probable cognitive impairment based on the Mini-Cog assessment (Supplementary Table 1).

Frailty category was associated with a higher Modified Medical Research Council (MMRC) dyspnea score and higher mean airway wall thickness (Pi10) on quantitative computed tomography (CT) scan across all respiratory subgroups (Table 2).

**Table 2:** Respiratory characteristics and exacerbations by frailty category.

		**All participants**				**Normal spirometry**				**GOLD 1**			
**(A)**		**Robust**	**Prefrail**	**Frail**	*p* ^*^	**Robust**	**Prefrail**	**Frail**	***p***	**Robust**	**Prefrail**	**Frail**	***p***
Characteristic	*N*	912	1352	401		473	589	108		115	154	27	
Age	2665	69 (7)	70 (8)	71 (9)	**<0.001**	68 (7)	69 (8)	71 (9)	**<0.001**	71 (8)	73 (8)	74 (10)	0.27
FEV1 % pred	2646	85 (22)	81 (24)	65 (28)	**<0.001**	99 (13)	100 (14)	97 (12)	0.20	92 (11)	92 (10)	92 (7)	0.57
Current Smoking	2663	26.2% (239)	34.2% (462)	34.4% (138)	**<0.001**	22.8% (108)	31.6% (186)	34% (37)	**0.003**	(31%) 36	(37%) 57	(52%) 14	0.13
Smoking Pack-Years	2663	39 (20)	43 (22)	53 (27)	**<0.001**	35 (18)	38 (21)	42 (21)	**0.003**	43 (22)	48 (24)	56 (30)	0.055
BODE score^†^	2645	0 (0, 1)	1 (0, 2)	4 (2, 6)	**<0.001**	0 (0, 0)	0 (0, 1)	2 (1, 3)	**<0.001**	0 (0, 0)	1 (0, 2)	3 (2, 5)	**<0.001**
MMRC score^†^	2663	0 (0, 1)	0 (0, 2)	3 (1, 3)	**<0.001**	0 (0, 1)	0 (0, 1)	1 (0, 3)	**<0.001**	0 (0, 1)	0 (0, 2)	2 (0, 3)	**<0.001**
Pi10	2433	2.16 (0.50)	2.28 (0.55)	2.64 (0.61)	**<0.001**	1.95 (0.40)	1.99 (0.41)	2.14 (0.43)	**<0.001**	2.08 (0.36)	2.16 (0.42)	2.47 (0.45)	**<0.001**
**(B)**													
Outcome	*N*	793	1122	307		407	489	80		104	127	21	
Annual exacerbation rate	2222	0.14 (0.50)	0.28 (0.75)	0.67 (1.46)	**<.001**	0.10 (0.43)	0.19 (0.71)	0.39 (1.48)	0.173	0.22 (0.68)	0.20 (0.57)	0.71 (2.56)	0.78
Any severe exacerbation	2222	7.8% (62)	13.4% (150)	26.1% (80)	**<.001**	4.4% (18)	7.4% (36)	15.0% (12)	**.002**	12.5% (13)	12.6% (16)	24% (5)	0.34
Frequent exacerbations	2222	3.4% (27)	9.4% (105)	22.1% (68)	**<.001**	2.0% (8)	6.1% (30)	11.3% (9)	**<.001**	5.8% (6)	7.1% (9)	10% (2)	0.74
		**GOLD 2–4**						**PRISm**					
		**Robust**		**Prefrail**		**Frail**	***p***	**Robust**		**Prefrail**		**Frail**	***p***
Characteristic		225		429		205		92		175		54	
Age		70 (7)		71 (8)		73 (9)	**0.008**	67 (8)		67 (8)		67 (8)	0.86
FEV1 % pred		59 (14)		56 (16)		43 (17)	**<0.001**	72 (8)		70 (8)		67 (10)	**0.01**
Current Smoking		27.6% (62)		32.9% (141)		30.7% (63)	0.38	34% (31)		44% (77)		39% (21)	0.26
Smoking Pack-Years		45 (20)		50 (23)		58 (27)	**<0.001**	40 (21)		38 (20)		52 (27)	**0.003**
BODE score^†^		1 (0, 3)		2 (1, 4)		6 (4, 7)	**<0.001**	0 (0, 1)		1 (0, 3)		3 (3, 5)	**<0.001**
MMRC score^†^		1 (0, 2)		2 (0, 3)		3 (3, 4)	**<0.001**	0 (0, 2)		1 (0, 3)		3 (2, 3)	**<0.001**
Pi10		2.54 (0.49)		2.69 (0.52)		2.91 (0.56)	**<0.001**	2.35 (0.50)		2.43 (0.50)		2.65 (0.55)	**0.003**
Outcome		198		358		160		78		145		42	
Annual exacerbation rate		0.18 (0.48)		0.45 (0.91)		0.88 (1.38)	**<.001**	0.18 (0.59)		0.20 (0.53)		0.42 (0.85)	**0.026**
Any severe exacerbation		12.6% (25)		22.6% (81)		33.1% (53)	**<.001**	8% (6)		11.7% (17)		21% (9)	0.087
Frequent exacerbations		4.0% (8)		15.9% (57)		31.9% (51)	**<.001**	6% (5)		6.2% (9)		12% (5)	0.44

(**A**) Cross-sectional respiratory characteristics. (**B**) Longitudinal follow-up exacerbations (mean follow-up time: 2.8 years). Note that 19 participants did not have spirometry data reported, and not all participants had longitudinal follow-up data (*N* reported separately for 2A and 2B). Continuous variables reported as mean (standard deviation) unless otherwise specified; categorical variables reported as % (*n*). Abbreviations: GOLD: Global Initiative for Chronic Obstructive Lung Disease; PRISm: Preserved Ratio Impaired Spirometry; FEV1 % pred: Forced expiratory volume in 1 second % predicted; BODE: Body mass index, airflow Obstruction, Dyspnea, and Exercise capacity; MMRC: Modified Medical Research Council Dyspnea Scale; Pi10: standardized airway wall thickness on quantitative CT scan (mm). ^*^p-value across frailty category (using Kruskal-Wallis rank sum test for continuous data, Pearson’s chi-squared test for categorical data with cell counts >5, and Fisher’s Exact test for categorical data with cell counts ≤5). ^†^Median (IQR).

In this population of current and former smokers, current smoking and smoking pack-years were associated with frailty category (Table 2), including among individuals with normal spirometry. In a combined model adjusted for age, sex, and forced expiratory volume in one second (FEV1) % predicted, the association with frailty in individuals with normal spirometry persisted for current smoking (Odds Ratio (OR) 2.8 (95% CI 1.7–4.8), *p* < 0.001) but not smoking pack-years.

In the post hoc analysis of the 249 never-smoker controls with frailty assessments (mean age = 67), 4 individuals (2%) were frail, and 84 individuals (34%) were prefrail (Supplementary Table 2).

### Distribution of frailty components

Shrinking and weakness were the most common features in the study cohort. Among frail individuals with moderate to very severe COPD, slowness and low activity were the most common (Supplementary Table 3 and Supplementary Figure 2). Principal Component Analysis (PCA) demonstrated cross-loading between low activity, slowness, and fatigue, and Multiple Correspondence Analysis (MCA) demonstrated contributions of low activity, slowness, and fatigue to the primary dimension (with which frailty was highly correlated) (Supplementary Figure 3).

### Respiratory characteristics and exacerbations

Exacerbation analyses included 2222 individuals with at least 180 days of follow-up (mean follow-up time = 2.8 years) (Supplementary Table 4 describes those without follow-up). Frail participants had significantly higher mean annual exacerbation rates compared to robust participants (0.67 events/year vs. 0.14 events/year, *p* < 0.001) and a higher unadjusted incidence of severe (26% vs. 8%, *p* < 0.001) and frequent (22% vs. 3%, *p* < 0.001) exacerbations (Table 2).

**Figure 1:**
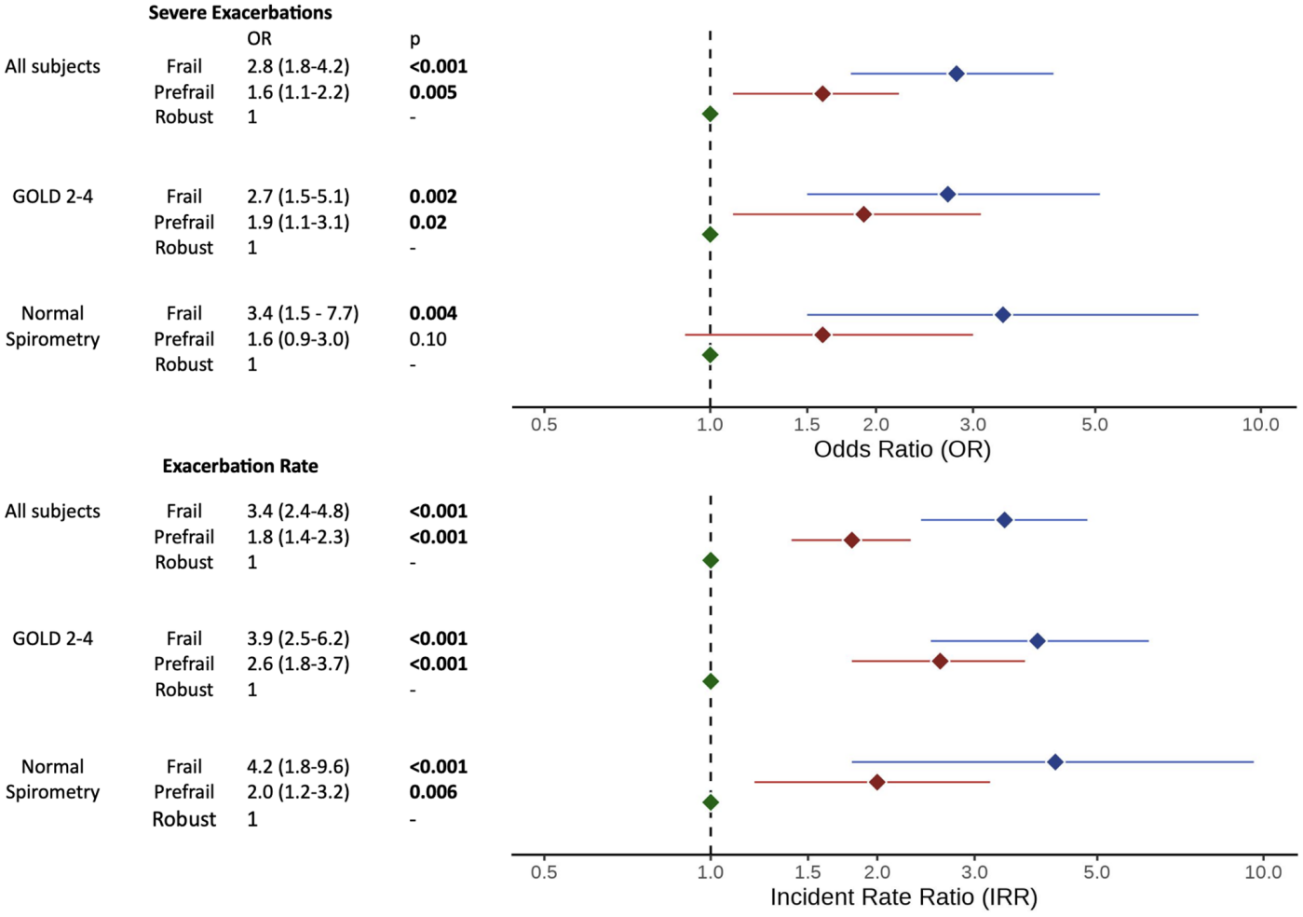
**Forest plot of frailty category on respiratory exacerbations.** Abbreviations: OR: odds ratio of frailty/prefrailty on severe exacerbations; IRR: incident rate ratio of frailty/prefrailty on annual exacerbation rate; GOLD: Global Initiative for Obstructive Lung Disease. OR/IRR and 95% confidence intervals (adjusted for age, sex, current smoking, and forced expiratory volume in one second (FEV1) %predicted) are shown on log-transformed x-axis. Full details in Supplementary Table 5.

In adjusted models, frailty was associated with increased exacerbation rate (Incidence Rate Ratio [IRR] 3.4 (95% CI 2.4–4.8), *p* < 0.001) and with increased odds of severe (OR 2.8 (1.8–4.2), *p* < 0.001) and frequent (OR 5.5 (3.2–9.3), *p* < 0.001) exacerbations (Figure 1 and Supplementary Table 5). Prefrailty was likewise associated with increased exacerbation rate (IRR 1.8 (1.4–2.3), *p* < 0.001), severe exacerbations (OR 1.6 (1.1–2.2), *p* = 0.005), and frequent exacerbations (OR 2.6 (1.7–4.1), *p* < 0.001). The frailty associations (and most prefrailty associations) persisted in subgroups analyses of those with moderate-very severe COPD and of those with normal spirometry (Figure 1). In the subgroups with fewer individuals (GOLD 1 and PRISm), associations between frailty and respiratory exacerbations did not consistently reach statistical thresholds, although effect estimates were in the same direction as in the overall analysis. Among individuals with PRISm, severe exacerbations were significantly associated with frailty (*p* = 0.047), and exacerbation rate had a trend towards association with frailty (*p* = 0.051) (Supplementary Table 5).

### Survival analysis - results

**Figure 2:**
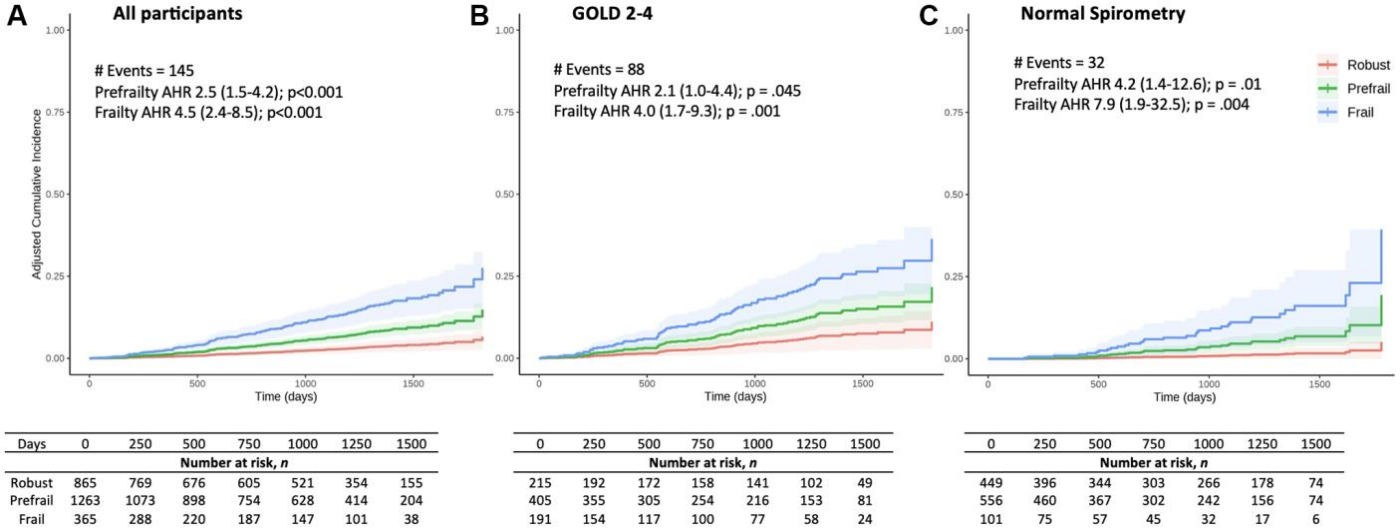
**Adjusted all-cause mortality cumulative incidence curves by frailty category.** Adjusted cumulative incidence (fraction) curves for (**A**) all participants, (**B**) individuals with GOLD 2–4 COPD, and (**C**) individuals with normal spirometry. The Cox adjusted Hazard Ratios (AHR) by frailty category (compared to robust group) are shown as: AHR (95% Confidence Interval); p-value. AHR was adjusted for age, sex, body mass index, smoking pack-years, FEV1 % predicted, diabetes, and heart disease (any of: coronary artery disease, myocardial infarction, angina, angioplasty, coronary artery bypass graft surgery, or congestive heart failure).

For the 2512 participants with mortality and covariate data (mean follow-up time = 2.6 years), adjusted risk curves of frailty and prefrailty on mortality are shown in Figure 2. Both frail (Adjusted Hazard Ratio (AHR) 4.5, 95% CI 2.4–8.5, *p* < 0.001) and pre-frail (AHR 2.5 (1.5–4.2), *p* < 0.001) individuals had an increased risk of death. These findings persisted in subgroup analyses of participants with GOLD 2–4 COPD (frailty AHR 4.0 (1.7–9.3), *p* = 0.001; prefrailty AHR 2.1 (1.02–4.4), *p* = 0.045) and with normal spirometry (frailty AHR 7.9 (1.9–32.5), *p* = 0.004; prefrailty HR 4.2 (1.4–12.6), *p* = 0.01). Adjusted survival analyses for the PRISm and GOLD 1 subgroups were not performed due to low event counts (details in Supplementary Table 6).

### Epigenetic pace of aging – results

Of 2104 subjects with DNA methylation data available at Phases 1 and 2 (Supplementary Table 7), analyses revealed associations between DunedinPACE of aging at Phase 1 and Phase 2 and frailty category (frail, prefrail, or robust) at Phase 3 (*p* < 0.001) (Supplementary Table 8 and Supplementary Figure 4). Associations persisted when stratified by smoking status at the time of blood draw, although unsurprisingly, individuals who were currently smoking tended to have higher DunedinPACE overall despite being chronologically younger (Supplementary Table 8 and Supplementary Figure 4). A sensitivity analysis of only those who did not report current smoking at Phase 1 nor at Phase 2 (“former-former” smoking) confirmed an association between DunedinPACE and frailty category. A sex-stratified sensitivity analysis of DunedinPACE on frailty status redemonstrated the association between DunedinPACE and frailty. Logistic regression demonstrated an association between baseline DunedinPACE and 10-year frailty (OR 2.8; 95% CI 2.3–3.4) and prefrailty (OR 1.9 (1.6–2.3)) **(**Supplementary Table 9 and Supplementary Figure 5). (Original DunedinPACE units were used, in which a value of one corresponds to one year of biological aging per year of chronological aging).

### Secondary analyses

There was no evidence of effect modification of FEV1 % predicted on the relationship between frailty and prefrailty and longitudinal outcomes.

Evaluation of the relationship between the number of frailty components on longitudinal outcomes demonstrated higher exacerbations and increased risk of death in individuals with more components present (Supplementary Figures 6, 7 and Supplementary Table 10).

In an adjusted Cox model evaluating all five frailty components together, shrinking, weakness, and slowness remained independently associated with mortality. In sex-stratified analyses, frailty remained associated with exacerbation and mortality outcomes for both men and women, although the effect estimates for women tended to be higher (Supplementary Table 11).

### Sensitivity analyses

When the frailty phenotype was operationalized using the slowness definition from the NETT trial [6], associations between frailty and prefrailty and primary outcomes (respiratory exacerbations and mortality) persisted. Two subgroup analyses (1. excluding individuals with probable cognitive impairment on the Mini-Cog, and 2. excluding those with body mass index (BMI) under 21) likewise demonstrated persistent associations between frailty and prefrailty and outcomes.

In an analysis excluding individuals who were frail or prefrail due to slowness (to rule out excessive influence of low six-minute walk distance (6MWD)), the associations between frailty and prefrailty and outcomes persisted. In analyses of the subgroup of only individuals who were frail or prefrail due to shrinking (since shrinking could represent successful dieting), frailty and prefrailty associations with mortality persisted, as did all frailty associations with exacerbation outcomes. Prefrailty’s association with some exacerbation outcomes attenuated in the subgroup of individuals with prefrailty due to shrinking.

We conducted a sensitivity analysis of respiratory exacerbation outcomes stratified by the timing of the Phase 3 visit (before or after the onset of the Covid-19 pandemic in March 2020). 28% of participants had visits after March 2020. Mean follow-up time was 3.4 years (pre-pandemic) vs. 1.3 years (post-pandemic). Frailty associations with exacerbations persisted in both the pre- and post-pandemic groups (Supplementary Table 12), although prefrailty findings lost statistical significance in the post-pandemic group. We were unable to perform a similar stratified analysis of mortality outcomes due to the small event number in the group whose site visit was post-pandemic.

## DISCUSSION

In this cohort of people with a history of cigarette smoking, frailty and prefrailty were prevalent regardless of spirometry. Current smoking status was associated with frailty, even among individuals with normal spirometry. The prevalence of frailty in our study cohort was within the range described in the literature; however, the frailty prevalence observed in post-hoc analysis of nonsmoker controls was low (2%), underscoring the connection between cigarette smoking and frailty.

Among participants 80 years of age or younger, age was not significantly associated with frailty, reinforcing that frailty is not simply a trait of chronologic aging. Frail individuals had increased need for support with activities of daily living and higher prevalence of probable cognitive impairment, highlighting the multi-system nature of this syndrome.

In models adjusted for lung function and smoking status/intensity, individuals with frailty had threefold higher exacerbation rates and fourfold higher hazard of death than robust individuals; findings persisted in subgroup analyses of individuals with normal spirometry and with moderate-very severe COPD.

The association between frailty and prospective respiratory exacerbations among people with normal spirometry has not to our knowledge been previously described. Frail individuals with normal spirometry also reported higher baseline dyspnea scores and had increased airway wall thickening, suggesting a potential inflammatory link between frailty and respiratory symptoms. These findings, combined with the emerging recognition of smoking-related respiratory pathology in people with normal spirometry, suggest that frailty should be considered in all people with a smoking history.

We did not observe significant associations between frailty and respiratory exacerbations among individuals with GOLD grade 1 COPD. Among the subgroup with PRISm, only severe exacerbations reached statistical significance for association with frailty, and a trend was observed for exacerbation rate. This may be related to the much smaller sample sizes in these two subgroups, in which we calculated lower power to detect differences. Further attention to these at-risk spirometric groups in follow-up studies is indicated.

This study underscores the risks associated with the prefrail state, as prefrail individuals had a roughly doubled exacerbation rates and increased mortality risk compared to robust individuals. On a more granular level, we identified that the presence of just one frailty component was associated with increased risk of adverse outcomes; which has previously been demonstrated for mortality but not for respiratory exacerbations [39].

These findings highlight the importance of recognizing frailty and prefrailty in the clinical setting and suggest a role for frailty screening in all adults with a smoking history, even those with normal spirometry. Improved recognition of prefrailty may inform earlier intervention points for preventing frailty, such as protein supplementation or nutritional counseling among prefrail individuals [40, 41]. Pulmonary rehabilitation has been associated with improvements in frailty status among individuals with COPD [6]; the potential benefits of pulmonary rehabilitation in pre-frail individuals may warrant investigation in future clinical studies.

Other metrics such as the BODE score have been used to predict outcomes in COPD [42]. While the Fried Frailty Phenotype (FFP) overlaps with some features of the BODE score, it evaluates for a distinct phenotype. For example, the modified FFP can identify frailty in individuals without airflow obstruction or with a BMI above 21 and is thus generalizable to a broader population. This is relevant as we found frailty and prefrailty in such individuals.

Frailty has been described as a state of physiologic dysregulation and disrupted homeostasis at a metabolic and cellular level, which leads to the observed phenotype [43]. In keeping with this, epigenetic associations between cigarette smoking and frailty have been identified [21]. In this study, we demonstrated that DunedinPACE, a novel metric of epigenetic aging, was associated with frailty status at 5- and 10-year follow-up. To our knowledge, this is the largest such analysis to be conducted and the first in a population enriched for a history of cigarette smoking. While baseline (Phase 1) frailty assessments were not performed, this adds to a recent prior study suggesting that an increased pace of aging could pre-date clinical frailty manifestations [38]. Despite the myriad effects of cigarette smoking on the epigenome, these findings were robust to stratification by current smoking status. Further research into the epigenetic underpinnings of frailty in populations with a smoking history could provide insight into disease mechanisms.

The strengths of our study include the large, well-phenotyped cohort and the presence of longitudinal follow-up for respiratory exacerbations and mortality. Its limitations include the length of follow-up time (which spanned the Covid-19 pandemic) and lack of cause-specific mortality data. Future research into respiratory-specific mortality related to frailty is needed. Furthermore, some spirometric subgroups had a low number of events, leading to widened confidence intervals of effect estimates for these subgroup analyses. As our study population had a history of smoking, the generalizability to never-smokers is unclear.

In conclusion, in a population of adults with a smoking history, frailty and prefrailty are associated with increased respiratory exacerbations and increased risk of death. The association between frailty and adverse outcomes is present in individuals with moderate to very severe COPD and in those with normal spirometry (and in PRISm for some exacerbation outcomes). Cigarette smoking was associated with frailty prevalence, even among those with normal spirometry. Frailty prevalence did not vary significantly with age among individuals under age 80. These findings highlight the importance of assessing for frailty and prefrailty in all adults with a history of smoking, even in those without advanced age and with normal spirometry.

## METHODS

### Study design and population

The COPDGene study (clinicaltrials.gov ID NCT00608764) is an ongoing multicenter cohort study [44]. Non-Hispanic White (NHW) and African American adults with a reported age 45–80 and a minimum 10 pack-year smoking history were eligible. Exclusion criteria included pulmonary fibrosis and active cancer under treatment. Participants had on-site evaluations at baseline (Phase 1) and every 5 years (Phases 2 and 3). All participants provided informed consent, and study protocols were approved by the institutional review board at each site.

**Figure 3:**
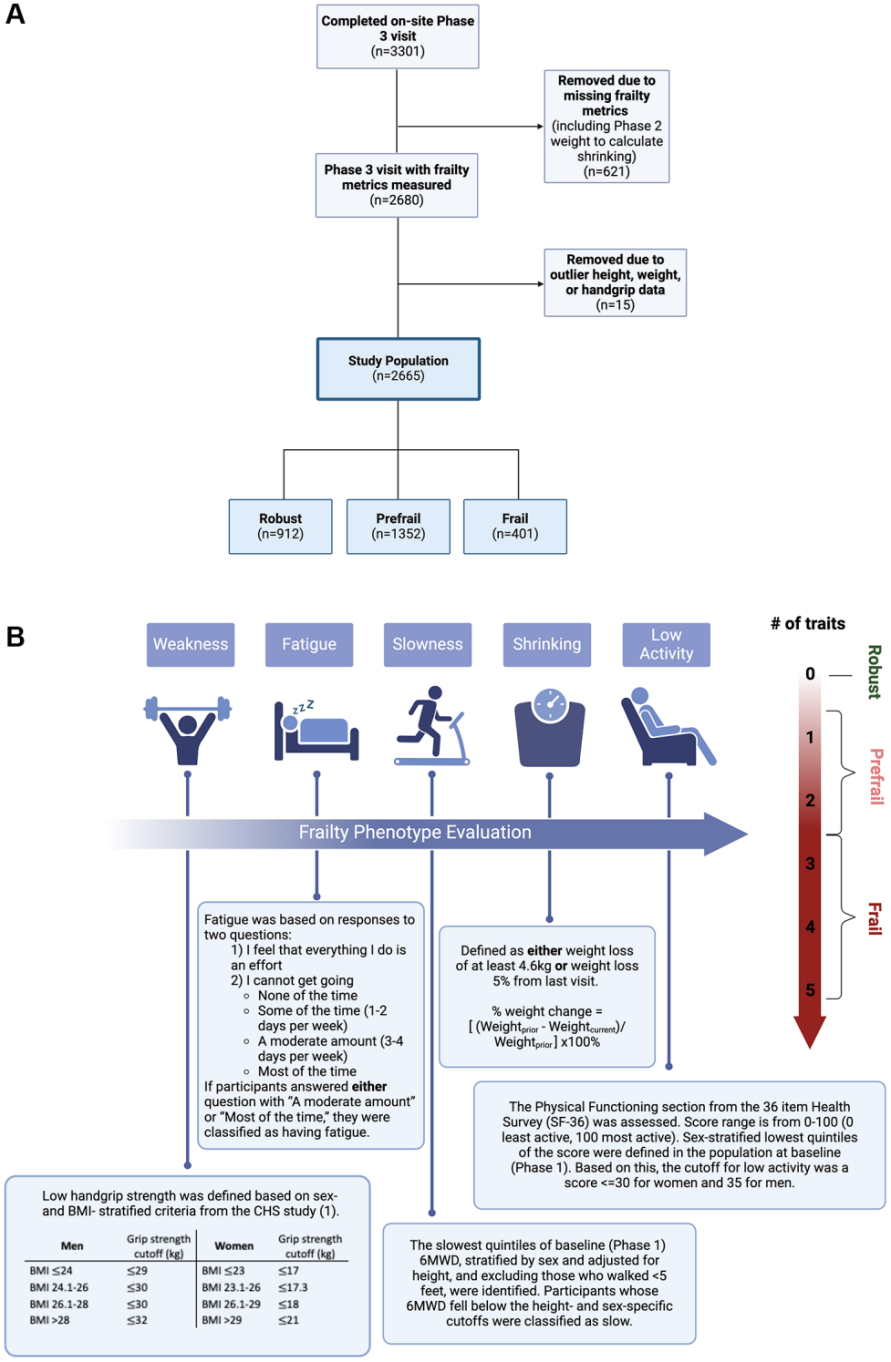
**Methods.** (**A**) CONSORT Diagram. (**B**) Frailty Phenotype Assessment. The frailty phenotype was evaluated based on five components: weakness, fatigue, slowness, shrinking, and low activity. Participants with three or more traits present were considered frail, those with one or two present were prefrail, and those without any traits present were classified as robust. Abbreviations: BMI: body mass index; 6MWD: six-minute walk distance; kg: kilograms; CHS: cardiovascular health study. Created in BioRender. Phillips, E. (2025) https://BioRender.com/6r3agi1 and https://BioRender.com/t42asu1.

The current study is an analysis of the COPDGene cohort limited to participants who returned for the Phase 3 (10-year follow-up) visit (2018–2023) and had an assessment of all five frailty components (Figure 3). Data were also collected on a smaller number of never-smoker controls; frailty prevalence was assessed in a post hoc analysis of this group.

### Measurements

Physiologic, spirometric, chest CT scan, and questionnaire data were collected by trained personnel at the Phase 3 visit. Hand grip strength (average of three efforts) was measured with Jamar dynamometers. Six-minute Walk tests (6MWT) were conducted in accordance with American Thoracic Society (ATS) guidelines [45]. Pre- and post-bronchodilator spirometry was performed using ndd EasyOne Spirometers (ndd Medical Technologies, Andover, MA, USA). Questionnaires included the 36-Item Short Form Survey (SF-36) and portions of the Center for Epidemiologic Studies Depression Scale (CES-D) [46, 47]. Additional details are in the Supplementary Methods.

We generated a modified FFP from the five frailty components: shrinking, weakness, low activity, fatigue, and slowness (Figure 3) [1]. Shrinking was defined as weight loss ≥4.6 kg or ≥5% of body weight from the prior (Phase 2) visit [1]; weakness was based on hand grip strength (with sex- and BMI-stratified cutoffs [1]); fatigue was assessed with standard questions from the CES-D; slowness was defined by the lowest quintile of 6MWD in the baseline population (adjusted for sex and height); low activity was defined by the sex-stratified lowest quintile of baseline SF-36 Physical Functioning scores. Defining frailty components by the lowest quintile in the baseline population is established in the literature [1, 18]. Individuals were classified as frail if three or more of these components were present, prefrail if one or two were present, and robust if none were present [1].

### Longitudinal outcome measurements

Longitudinal follow-up on respiratory exacerbations was collected at six-month intervals by telephone or web-based survey. Exacerbation data and unadjudicated all-cause mortality data are reported through July 2023.

Participants with fewer than 180 days of follow-up were excluded from exacerbation analyses. Exacerbations were defined as an episode of increased cough and phlegm or shortness of breath which lasted for at least 48 hours and required treatment with antibiotics, steroids, emergency room (ER) visit, or hospitalization. We evaluated annual exacerbation rate, presence of severe exacerbations, and presence of frequent exacerbations (defined in Supplementary Methods).

### Epigenetic pace of aging measurement

Whole blood samples for assessment of DNA methylation were obtained at baseline (Phase 1) visit and at 5-year follow-up (Phase 2) visit. DNA methylation was assessed using the Illumina Infinium EPIC 850 k BeadChip array. After regression on correlated probes for bias correction and functional normalization, methylation beta values were used to calculate the DunedinPACE of Aging using the DunedinPACE package in R statistical software. Individuals who were missing either Phase 1 or Phase 2 methylation data were excluded from epigenetic pace of aging analyses.

### Statistical analysis

Continuous variables are reported as mean (standard deviation) unless specified. Differences across frailty categories were assessed with Kruskal-Wallis rank sum test, Pearson’s chi-squared test, and Fisher’s Exact test.

To evaluate how the five frailty components combined to generate the frailty phenotype, we performed a PCA of the five continuous characteristics from which frailty components were derived and a complementary MCA of the five binary traits.

For longitudinal outcomes analyses, robust individuals were used as the comparator group for frail and prefrail individuals. Frequent and severe exacerbations were modeled using multivariable logistic regression. Exacerbation rate was modeled by multivariable negative binomial regression of total exacerbation count with an offset term of log(follow-up time) [48]. Exacerbation models were adjusted for participant age, sex, % predicted post-bronchodilator forced expiratory volume in 1 second (FEV1), and smoking status.

Multivariable Cox proportional hazards models adjusted for a priori covariates of age, sex, BMI, smoking pack-years, diabetes, and heart disease (defined in Supplementary Methods) were used to calculate adjusted hazard ratios (AHR) for frailty and prefrailty.

Subgroup analyses were performed by spirometric category (definitions in Supplementary Methods): normal spirometry, GOLD 1, GOLD 2–4, and PRISm. Secondary and sensitivity analyses (including evaluation of the interaction term between FEV1 %predicted and frailty on outcomes and evaluation of outcomes by the number of frailty components) are described in the Supplementary Methods.

The frequency of missing cross-sectional covariate data is reported, as are characteristics of subjects without longitudinal follow-up data and of those without epigenetic pace of aging data. In cases of missing data, complete case analysis was performed.

The association between epigenetic pace of aging (DunedinPACE) at baseline (Phase 1) and at 5-year follow-up (Phase 2) and frailty status at 10-year follow-up (Phase 3) was assessed. To evaluate for potential confounding epigenetic effects of current smoking, analyses were stratified by smoking status at the time of blood sample collection. Crude associations were assessed using Kruskal-Wallis rank sum test. Logistic regressions of Phase 1 and Phase 2 DunedinPACE of Aging on the outcome of Phase 3 frailty (vs. robustness) and prefrailty (vs. robustness) were performed. One unit of DunedinPACE can be interpreted as one year of biological aging per year of chronological aging; medians and interquartile ranges of these values (stratified by Phase and smoking status) are reported. A sensitivity analysis evaluating only former-former smokers (former at both Phase 1 and Phase 2) was conducted, as was a sensitivity analysis comparing DunedinPACE with frailty status when stratified by sex.

Statistical analyses were conducted in R 4.3.0 (with the exception of the calculation of DunedinPACE, which was conducted in R 4.2.0).

## Supplementary Materials

Supplementary Methods

Supplementary Figures

Supplementary Tables
