# Supplementary material for: Frailty associates with respiratory exacerbations and mortality in the COPDGene cohort
Source: Aging (Albany NY). 2025 Jul 3;17(7):1590–623. doi: 10.18632/aging.206275 (PMC12339021; doi:10.18632/aging.206275)
Supplement: Supplementary Methods [file aging-17-206275-s001.pdf]

SUPPLEMENTARY METHODS

Study design and population

The COPDGene study (clinicaltrials.gov ID NCT00608764) is an ongoing multicenter cohort study [1]. Non-Hispanic White and African American adults with a reported age 45–80 and a minimum 10 pack-year smoking history were eligible to participate. Exclusion criteria included pulmonary fibrosis, active cancer under treatment, and history of chest radiation. Participants had on-site evaluations at baseline (Phase 1) and every 5 years (Phases 2 and 3). Participants provided written documentation of the informed consent process, and study protocols were approved by the institutional review board of each clinical center.

The current study is an analysis of the COPDGene cohort limited to the participants who returned for the Phase 3 (10-year follow-up) visit (2018–2023) and had an assessment of all five frailty components (Figure 3). Data were also collected on a small number of never-smoker controls; frailty prevalence was assessed in a post hoc analysis of this group.

Measurements

Physiologic, spirometric, questionnaire, and CT data were collected by trained personnel at the Phase 3 visit. Hand grip strength (average of three efforts) was measured with Jamar dynamometers. Six-minute walk tests (6MWT) were conducted in accordance with ATS guidelines [2].

Pre- and post-bronchodilator spirometry was performed using ndd EasyOne Spirometers. Post-bronchodilator forced expiratory volume in one second (FEV1) % predicted was defined based on NHANES III references [3]. COPD was defined as an FEV1/FVC (forced vital capacity) ratio of <0.7, and GOLD grade was defined according to standard criteria [4]. Preserved Ratio Impaired Spirometry (PRISm) was defined as an FEV1 of <80% predicted and an FEV1/FVC ratio of >0.7 [5].

Questionnaires included the 36-Item Short Form Survey (SF-36) and portions of the Center for Epidemiologic Studies Depression Scale (CES-D) [6, 7].

Two self-reported questions about need for assistance with basic and instrumental activities of daily living (ADLs) were asked based on recommendations by the Alzheimer's Association's Medicare Detection of Cognitive Impairment Workgroup [8]:

(1) "During the past 7 days, did you need help from others to perform everyday activities such as eating, getting dressed, grooming, bathing, walking, or using the toilet?" (Yes/No)

(2) "During the past 7 days, did you need help from others to take care of things such as laundry and housekeeping, banking, shopping, using the telephone, food preparation, transportation, or taking your own medications?" (Yes/No)

The Mini-Cog was administered as a brief standardized screen for cognitive impairment, with probable cognitive impairment defined as a total score ≤3. This cutoff was chosen based on prior literature examining the Mini-Cog sensitivity and specificity relative to the gold standard assessment [9, 10].

Comorbidities and smoking status were self-reported by survey. For this study, comorbidity count was defined as the sum of the following: congestive heart failure, coronary artery disease (CAD) composite, cerebrovascular disease, kidney disease, liver disease, diabetes, osteoarthritis, osteoporosis, and cancer (excluding non-melanoma skin cancers). CAD (composite) was defined by the presence of any of the following: self-reported CAD, myocardial infarction, angina, angioplasty, or coronary artery bypass graft surgery. Heart disease (composite) was defined if participants reported any of the following: CAD, myocardial infarction, angina, angioplasty, coronary artery bypass graft surgery, or congestive heart failure. Cerebrovascular disease (composite) was defined as the presence of reported stroke and/or transient ischemic attack (TIA).

Detailed protocols regarding CT scan data collection and analysis have been described previously [1]. Volumetric CT acquisitions were obtained, and images were reconstructed using sub-millimeter slice thickness. Quantitative CT measurement of the standardized airway wall thickness (Pi10) was calculated using Thirona software based on the average wall thickness of a hypothetical airway with a lumen perimeter of 10mm.

Frailty assessment

We generated a modified FFP from the five frailty components of shrinking, weakness, low activity, fatigue, and slowness (Figure 1) [11].

Shrinking was defined as weight loss ≥4.6kg or ≥5% of body weight from the prior (Phase 2) visit [11].

Weakness was based on hand grip strength (with sex- and body mass index (BMI)-stratified cutoffs [11]).

Fatigue was assessed with two questions from the CES-D:

(1) "I feel that everything I do is an effort"
(2) "I cannot get going"

Response options were "None of the time", "Some of the time (1–2 days per week)", "A moderate amount (3–4 days per week)", and "Most of the time." Individuals who answered "A moderate amount (3–4 days per week)" or "Most of the time" to either question were considered to have fatigue.

Slowness was defined by the lowest quintile of 6MWD in the baseline (Phase 1) population (stratified by sex and adjusted for height). Defining frailty components by the lowest quintile in the baseline population is established in the literature [11, 12].

Low activity was based on responses to the SF-36 survey Physical Functioning (PF) section. This section consists of 10 questions as follows:

"The following questions are about activities you might do during a typical day. Does your health now limit you in these activities? If so, how much?

(a) Vigorous activities, such as running, lifting heavy objects, participating in strenuous sports
(b) Moderate activities, such as moving a table, pushing a vacuum cleaner, bowling, or playing golf
(c) Lifting or carrying groceries
(d) Climbing several flights of stairs
(e) Climbing one flight of stairs
(f) Bending, kneeling, or stooping
(g) Walking more than a mile
(h) Walking several hundred yards
(i) Walking one hundred yards
(j) Bathing or dressing yourself"

SF-36 PF response options were "Yes, limited a lot", "Yes, limited a little", and "No, not limited at all". Responses were scored 0 (not limited), 50 (somewhat limited), or 100 (limited a lot), and the mean of the responses was used as the PF section score [6]. Low activity was defined by the sex-stratified lowest quintile of baseline (Phase 1) SF-36 PF scores.

Individuals were classified as frail if three or more of these components were present, prefrail if one or two were present, and robust if none were present [11].

Longitudinal outcome measurements

Longitudinal follow-up on respiratory exacerbations was collected at six-month intervals by telephone or web-based survey. Exacerbation data and unadjudicated all-cause mortality data are reported through July 2023.

Participants with fewer than 180 days of follow-up were excluded from exacerbation analyses. Exacerbations were defined as an episode of increased cough and phlegm or shortness of breath which lasted for at least 48 hours and which required treatment with antibiotics, steroids, emergency room (ER) visit, or hospitalization. Exacerbations requiring an ER visit or hospitalization were classified as severe. The exacerbation outcomes evaluated were annual exacerbation rate, presence of severe exacerbations, and presence of frequent exacerbations (annual rate of ≥1/year).

Epigenetic pace of aging measurement

Whole blood samples for assessment of DNA methylation were obtained at baseline (Phase 1) visit and at 5-year follow-up (Phase 2) visit. DNA methylation was assessed using the Illumina Infinium EPIC 850k BeadChip array. After regression on correlated probes for bias correction and functional normalization, methylation beta values were used to calculate the DunedinPACE of Aging using the DunedinPACE package in R statistical software. Individuals who were missing either Phase 1 or Phase 2 methylation data were excluded from epigenetic pace of aging analyses.

Statistical analysis

Continuous variables are reported as mean (standard deviation) unless otherwise specified. Differences across frailty categories were assessed with Kruskal-Wallis rank sum test (continuous data), Pearson's chi-squared test (categorical data), and Fisher's exact test (categorical data with cell counts ≤5).

To evaluate how the five frailty components combined to generate the frailty phenotype, we performed a principal component analysis (PCA) of the five continuous underlying characteristics from which frailty traits were derived. PCA was performed on scaled data using the variance-covariance method. A complementary multiple correspondence analysis (MCA) of the five binary frailty traits was conducted using an indicator matrix.

For analyses of exacerbation data, robust individuals were used as the comparator group for frail and prefrail individuals. Frequent and severe exacerbations were

modeled using multivariable logistic regression. Exacerbation count was analyzed using multivariable negative binomial regression of total exacerbation count with an offset term for the log(follow-up time) [13]. Exacerbation models were adjusted for participant age, sex, % predicted post-bronchodilator forced expiratory volume in 1 second (FEV1), and smoking status.

Multivariable Cox proportional hazard models adjusted for a priori covariates of age, sex, BMI, smoking pack-years, diabetes, and heart disease (defined above) were used to calculate adjusted hazard ratios (AHR) for frailty and prefrailty. Robust individuals were used as the comparison group. Linearity was tested using likelihood ratios comparing the model used to model with covariate terms including second order polynomials. A sensitivity analysis was performed using a parametric (Weibull) multivariable model to confirm persistence of frailty and prefrailty effects. Covariates were selected based on factors that had been (clinically or scientifically) associated with both frailty and mortality and thus could potentially confound analyses. For this reason, covariate selection differed slightly between exacerbation and mortality analyses (for example, diabetes was included in the model for mortality but not in those for respiratory exacerbations). Adjusted cumulative incidence curves were obtained using the G-formula method [14].

Subgroup analyses were performed by spirometric category (normal spirometry, GOLD 1, GOLD 2–4, and PRISm; defined above), with a focus on the two subgroups with the largest number of participants (normal spirometry and GOLD 2–4).

The association between epigenetic pace of aging (DunedinPACE) at baseline (Phase 1) and at 5-year follow-up (Phase 2) and frailty status at 10-year follow-up (Phase 3) was assessed. To evaluate for potential confounding epigenetic effects of current smoking, analyses were stratified by smoking status at the time of blood sample collection. Crude associations were assessed using Kruskal-Wallis rank sum test. Logistic regressions of Phase 1 and Phase 2 DunedinPACE of Aging on the outcome of Phase 3 frailty (vs robustness) and prefrailty (vs robustness) were performed. One unit of DunedinPACE can be interpreted as one year of biological aging per year of chronological aging; medians and interquartile ranges of these values (stratified by Phase and smoking status) are reported.

Statistical analyses were conducted in R 4.3.0 (with the exception of the calculation of DunedinPACE, which was conducted in R 4.2.0). Software packages used included survival, adjustedCurves, methylCIPHER, and FactoMineR. A two-sided *p*-value of <0.05 was considered statistically significant unless otherwise specified.

Missing data

The frequency of missing cross-sectional covariate data is reported. Characteristics of subjects with and without missing follow-up and methylation data are reported. In cases of missing data in regression models, complete case analysis was performed.

Secondary analyses

To explore the observed cross-sectional associations between cigarette smoking and frailty category, we performed exploratory post-hoc multivariable logistic regressions of outcomes of frailty and prefrailty against covariates of age, sex, FEV1 % predicted, smoking status, and smoking pack-years. The purpose of this was to evaluate if the association between frailty and prefrailty and smoking persisted after adjusting for age and lung function. These were performed on the entire study population and for each spirometric subgroup (normal spirometry, GOLD 1, GOLD 2–4, and PRISm).

For longitudinal outcomes, we evaluated for effect modification of lung function on frailty/prefrailty's association with outcomes by adding an interaction term for FEV1% predicted * frailty (or FEV1 % predicted *prefrailty) to above models.

We also evaluated the association between the number of frailty traits (0–5, categorical) and respiratory exacerbations (exacerbation rate, severe exacerbations, and frequent exacerbations) and with mortality (collapsing the groups with 4 and 5 traits due to low event counts).

To assess if any one frailty component was overly influential to mortality risk, we separately performed a Cox proportional hazard model (adjusted for covariates in primary mortality model) including all five individual frailty components (instead of frailty category) as predictors.

We also performed sex-stratified analyses of the associations between prefrailty and frailty and longitudinal outcomes.

Lastly, we characterized the prevalence of frailty and prefrailty in the non-smoker control group.

Sensitivity analyses

We performed several sensitivity analyses of the associations between frailty and prefrailty and the

primary outcomes of exacerbations and mortality (of the primary effects only; not respiratory subgroup analyses).

(1) We used an alternative frailty and prefrailty definition based on the slowness criteria used in the NETT trial, which defined slowness as a 6MWD of ≤770 feet for men 173 cm or shorter and women 159cm or shorter, and otherwise as a 6MWD of ≤900 feet [15].
(2) We excluded individuals with a Mini-Cog score of 3 or lower from analyses (since frailty status ascertainment involved self-reported measures).
(3) To assess for overly influential effects of underweight subjects, we performed a subgroup analysis on only those with BMI over 21.
(4) To confirm that frailty effects were not simply driven by low 6MWD, we *excluded* individuals who were frail due to slowness (that is, had exactly 3 frailty components, one of which was slowness) or who were prefrail due to slowness (that is, had only one frailty component: slowness).
(5) We *looked only at the subgroup* of individuals who were frail and prefrail due to shrinking to see if associations with adverse outcomes persisted in this group as well (since successful dieting could be classified as shrinking). Individuals who were considered frail "due to shrinking" had exactly three frailty components, one of which was shrinking. Individuals who were prefrail "due to shrinking" had only one frailty component present (shrinking).
(6) We performed stratified analyses based on whether participants had their Phase 3 visit before or after 2020 to assess for pandemic effects.

For epigenetic pace of aging analyses, a sensitivity analysis evaluating only former-former smokers (former at both Phase 1 and Phase 2) was conducted, as was a sensitivity analysis comparing DunedinPACE with frailty status when stratified by sex.

SUPPLEMENTARY REFERENCES

1. Regan EA, Hokanson JE, Murphy JR, Make B, Lynch DA, Beaty TH, Curran-Everett D, Silverman EK, Crapo JD. Genetic epidemiology of COPD (COPDGene) study design. COPD. 2010; 7:32–43. https://doi.org/10.3109/15412550903499522 PMID:20214461

2. ATS Committee on Proficiency Standards for Clinical Pulmonary Function Laboratories. ATS statement: guidelines for the six-minute walk test. Am J Respir Crit Care Med. 2002; 166:111–7. https://doi.org/10.1164/ajrccm.166.1.at1102 PMID:12091180

3. Hankinson JL, Odencrantz JR, Fedan KB. Spirometric reference values from a sample of the general U.S. population. Am J Respir Crit Care Med. 1999; 159:179–87. https://doi.org/10.1164/ajrccm.159.1.9712108 PMID:9872837

4. Agustí A, Celli BR, Criner GJ, Halpin D, Anzueto A, Barnes P, Bourbeau J, Han MK, Martinez FJ, Montes de Oca M, Mortimer K, Papi A, Pavord I, et al. Global Initiative for Chronic Obstructive Lung Disease 2023 Report: GOLD Executive Summary. Eur Respir J. 2023; 61:2300239. https://doi.org/10.1183/13993003.00239-2023 PMID:36858443

5. Wan ES, Fortis S, Regan EA, Hokanson J, Han MK, Casaburi R, Make BJ, Crapo JD, DeMeo DL, Silverman EK, and COPDGene Investigators. Longitudinal Phenotypes and Mortality in Preserved Ratio Impaired Spirometry in the COPDGene Study. Am J Respir Crit Care Med. 2018; 198:1397–405. https://doi.org/10.1164/rccm.201804-0663OC PMID:29874098

6. Ware JE Jr. SF-36 health survey update. Spine (Phila Pa 1976). 2000; 25:3130–9. https://doi.org/10.1097/00007632-200012150-00008 PMID:11124729

7. Radloff LS. The CES-D Scale: A Self-Report Depression Scale for Research in the General Population. Appl Psychol Meas. 1977; 1:385–401. https://hdl.handle.net/11299/98561.

8. Cordell CB, Borson S, Boustani M, Chodosh J, Reuben D, Verghese J, Thies W, Fried LB, and Medicare Detection of Cognitive Impairment Workgroup. Alzheimer's Association recommendations for operationalizing the detection of cognitive impairment during the Medicare Annual Wellness Visit in a primary care setting. Alzheimers Dement. 2013; 9:141–50. https://doi.org/10.1016/j.jalz.2012.09.011 PMID:23265826

9. Borson S, Scanlan JM, Chen P, Ganguli M. The Mini-Cog as a screen for dementia: validation in a population-based sample. J Am Geriatr Soc. 2003; 51:1451–4. https://doi.org/10.1046/j.1532-5415.2003.51465.x PMID:14511167

10. Borson S, Scanlan J, Brush M, Vitaliano P, Dokmak A. The mini-cog: a cognitive 'vital signs' measure for dementia screening in multi-lingual elderly. Int J Geriatr Psychiatry. 2000; 15:1021–7. https://doi.org/10.1002/1099-1166(200011)15:11<1021::aid-gps234>3.0.co;2-6 PMID:11113982

11. Fried LP, Tangen CM, Walston J, Newman AB, Hirsch C, Gottdiener J, Seeman T, Tracy R, Kop WJ, Burke G, McBurnie MA, and Cardiovascular Health Study Collaborative Research Group. Frailty in older adults: evidence for a phenotype. J Gerontol A Biol Sci Med Sci. 2001; 56:M146–56. https://doi.org/10.1093/gerona/56.3.m146 PMID:11253156

12. Bandeen-Roche K, Seplaki CL, Huang J, Buta B, Kalyani RR, Varadhan R, Xue QL, Walston JD, Kasper JD. Frailty in Older Adults: A Nationally Representative Profile in the United States. J Gerontol A Biol Sci Med Sci. 2015; 70:1427–34. https://doi.org/10.1093/gerona/glv133 PMID:26297656

13. Gardner W, Mulvey EP, Shaw EC. Regression analyses of counts and rates: Poisson, overdispersed Poisson, and negative binomial models. Psychol Bull. 1995; 118:392–404. https://doi.org/10.1037/0033-2909.118.3.392 PMID:7501743

14. Denz R, Klaaßen-Mielke R, Timmesfeld N. A comparison of different methods to adjust survival curves for confounders. Stat Med. 2023; 42:1461–79. https://doi.org/10.1002/sim.9681 PMID:36748630

15. Kennedy CC, Novotny PJ, LeBrasseur NK, Wise RA, Sciurba FC, Benzo RP. Frailty and Clinical Outcomes in Chronic Obstructive Pulmonary Disease. Ann Am Thorac Soc. 2019; 16:217–24. https://doi.org/10.1513/AnnalsATS.201803-175OC PMID:30433830
