# Supplementary material for: Frailty associates with respiratory exacerbations and mortality in the COPDGene cohort
Source: Aging (Albany NY). 2025 Jul 3;17(7):1590–623. doi: 10.18632/aging.206275 (PMC12339021; doi:10.18632/aging.206275)
Supplement: Supplementary Figures [file aging-17-206275-s002.pdf]

SUPPLEMENTARY FIGURES

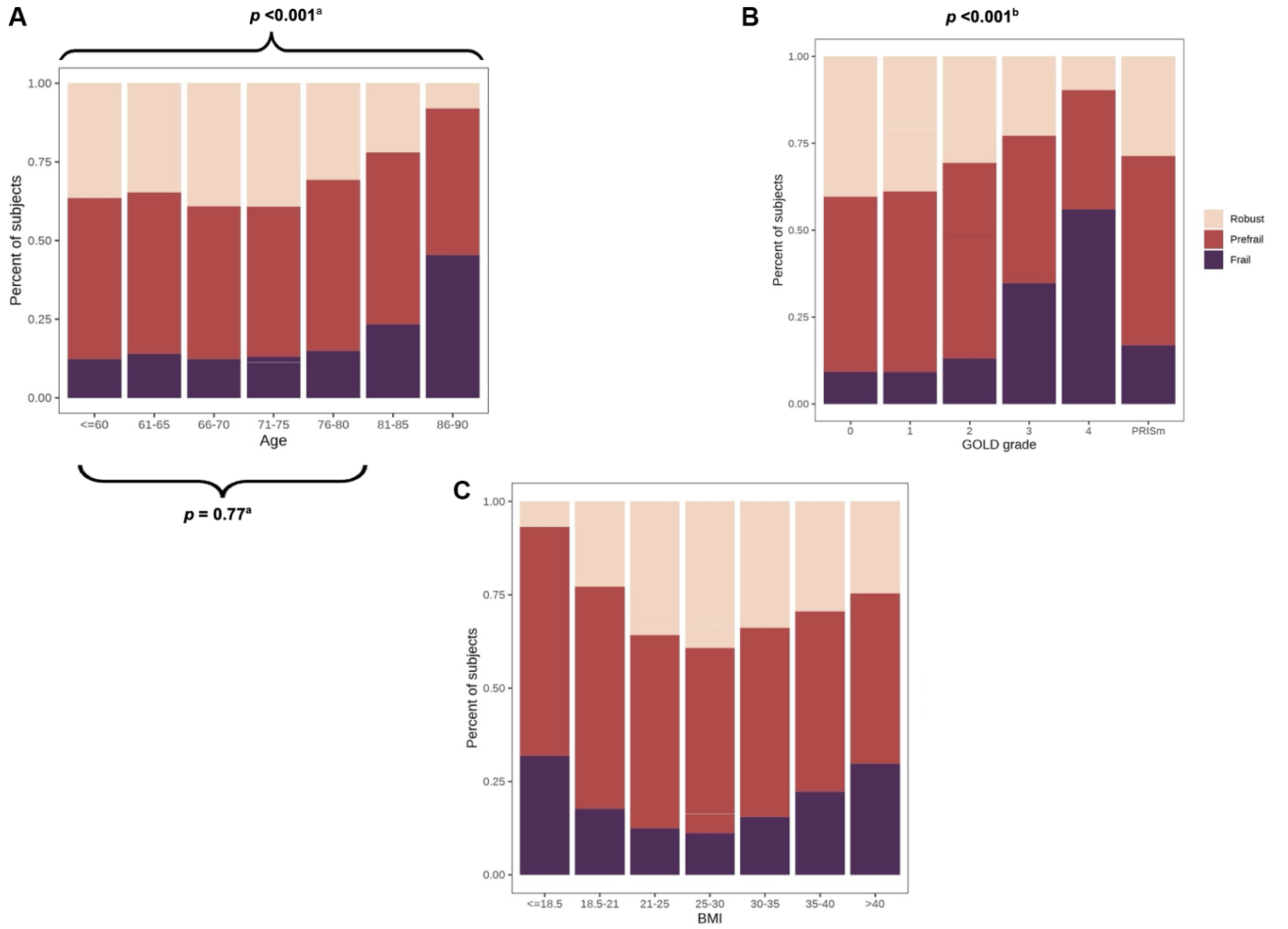

Supplementary Figure 1. Frailty prevalence by age group, GOLD grade, and BMI category. Stacked bar graphs demonstrating the prevalence of frailty category (frail, prefrail, and robust) (**A**) across age categories (**B**) within each GOLD grade and (**C**) by BMI stratum. [a]*p*-values by Kruskal-Wallis rank sum test of participant age (continuous) across frailty category (frail, prefrail, robust) for all subjects (upper) and for subjects aged 80 or younger (lower). [b]Pearson's chi-squared *P*-value across all 6 categories (GOLD 0–4 and PRISm) shown. Abbreviations: GOLD: Global Initiative for Chronic Obstructive Lung Disease; PRISm: Preserved Ratio Impaired Spirometry; BMI: Body Mass Index (kg/m^2).

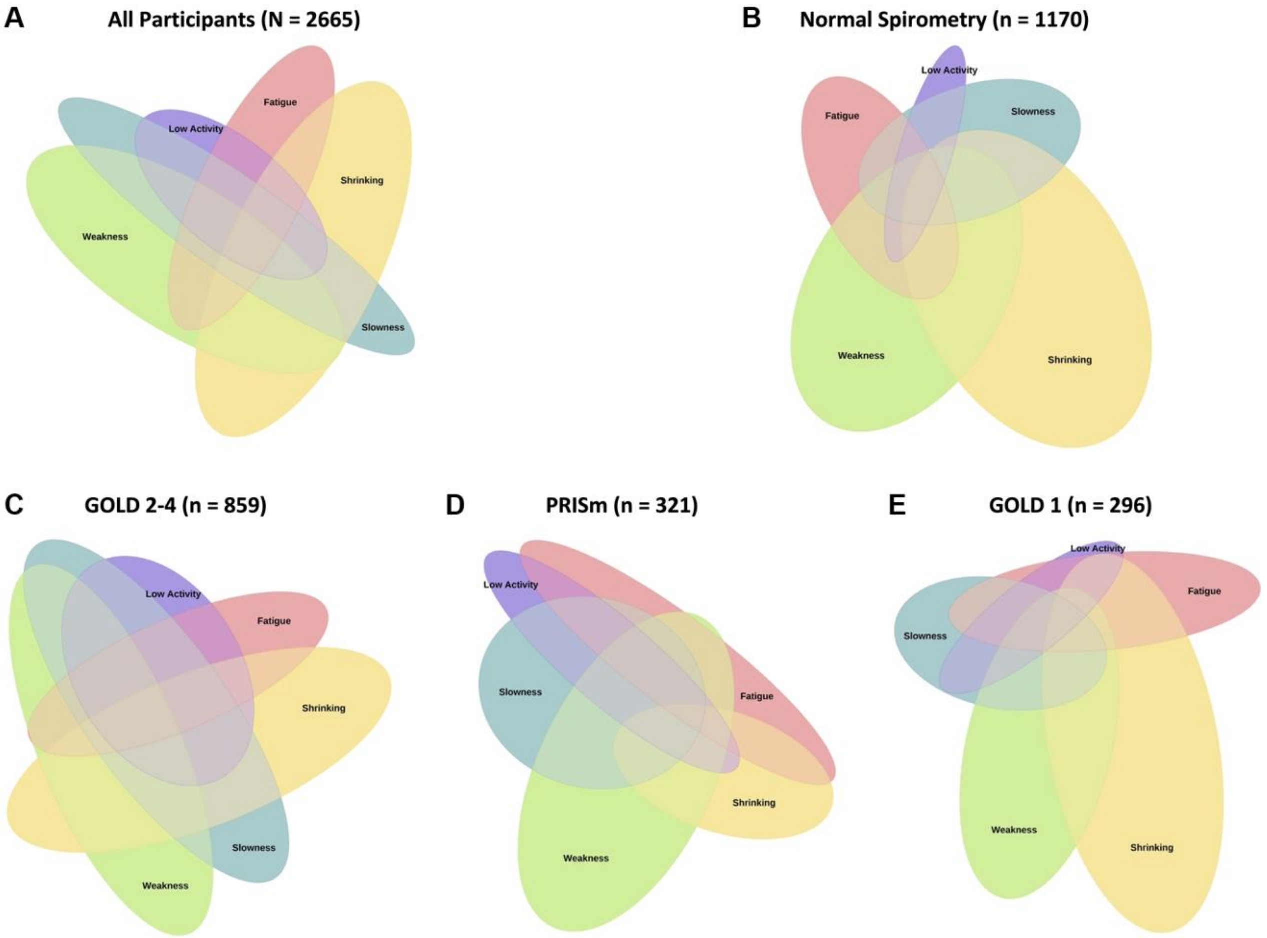

Supplementary Figure 2. Loading of frailty components. Proportional Euler diagrams of frailty components, (**A**) for all participants and (**B**–**E**) by spirometric category (in order of group size). Participants with 3 or more components present were frail, those with one or two present were prefrail. Number of participants with missing spirometry = 19. Abbreviation: GOLD: Global Initiative for Chronic Obstructive Lung Disease.

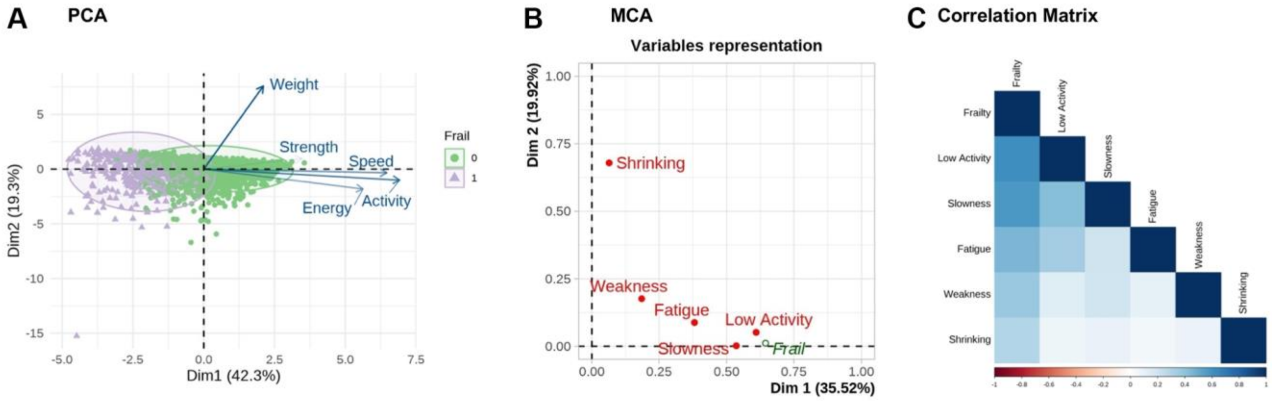

All subjects

	PC 1	PC 2	PC 3	PC 4	PC 5
Eigenvalue	2.1	.97	.93	.63	.36
Variance %	42	19	19	13	7
Cum. var. %	42	62	80	93	100
Speed	.80	-.04	.08		
Activity	.86	-.13	-.15		
Energy	.70	-.23	-.36		
Weight	.26	.94	-.21		
Strength	.43	.12	.86		

Supplementary Figure 3. Correlation of frailty characteristics. (**A**) Principal Component Analysis (PCA) biplot with table of eigenvalues and principal component loadings. The intensity of a variable (arrow) color is based on the strength of its contribution. Speed: 6-minute walk distance, Activity: 36-Item Short Form Survey Physical Function Score, Energy: inverse score of fatigue questions on the Center for Epidemiologic Studies Depression Scale (CES-D), Strength: grip strength (kilograms), Weight: inverse weight loss (or 0 if weight gain). Note that for the underlying characteristics, higher levels indicate a more robust status. Cum. var. %: cumulative variance %. (**B**) Multiple Correspondence Analysis (MCA) variable map of individual frailty traits (red) and the supplementary variable of overall frailty (green) against the two principal dimensions. (**C**) Correlation matrix of frailty components (degree of shading is Pearson's correlation coefficient between components).

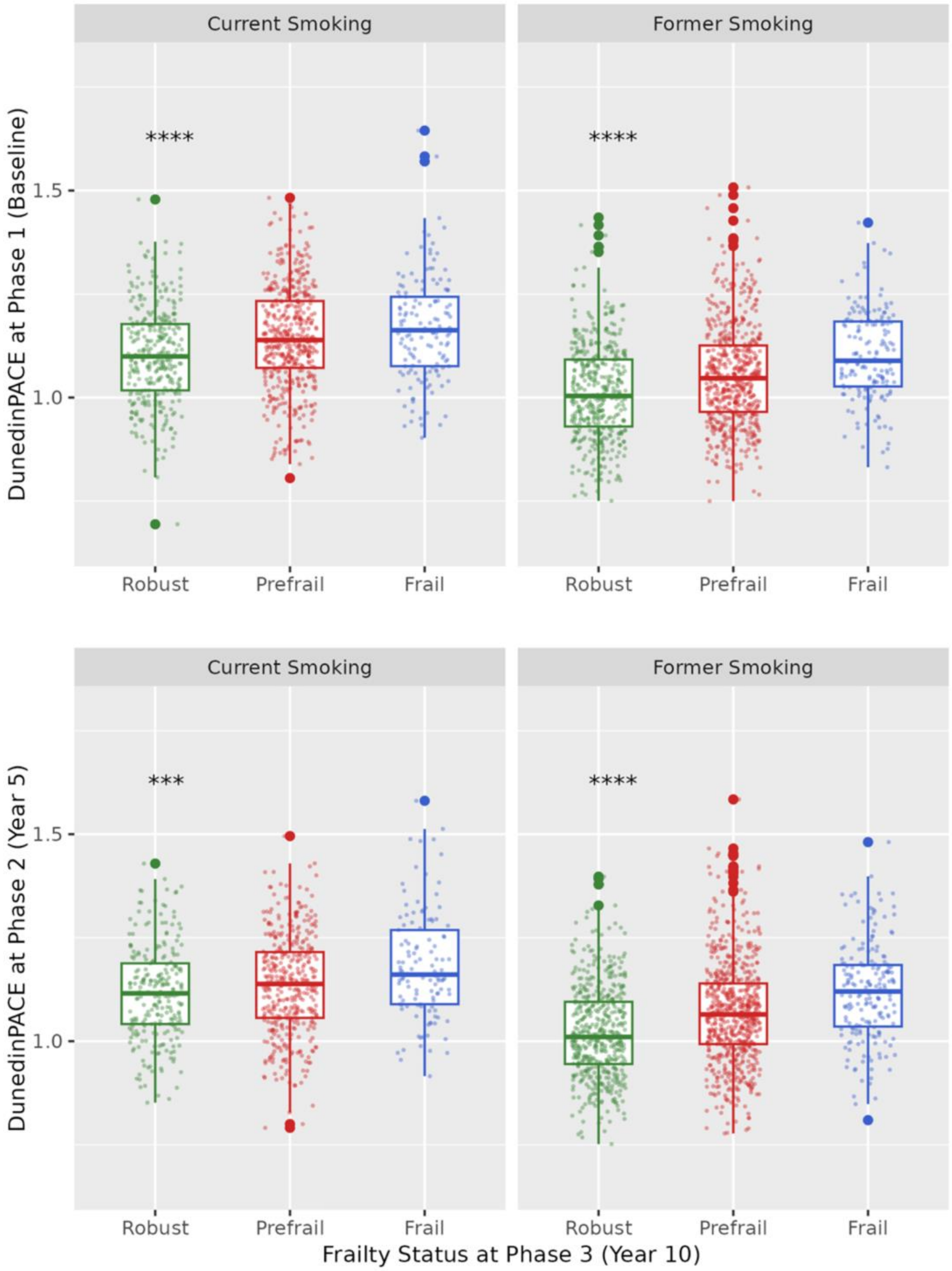

Supplementary Figure 4. Scatter plot of DunedinPACE (Phase 1 and Phase 2) by frailty status (Phase 3). DunedinPACE values at Phase 1 (baseline, top) and Phase 2 (5-year follow-up, bottom), stratified by smoking status (current, left; former, right) are displayed by frailty category at Phase 3 (10-year follow-up). Stars indicate Kruskal-Wallis rank sum test *p*-value across frailty category for that phase and stratum ($^{***}p < .001$, $^{****}p < .0001$). Tabular format of this data is in Supplementary Table 8.

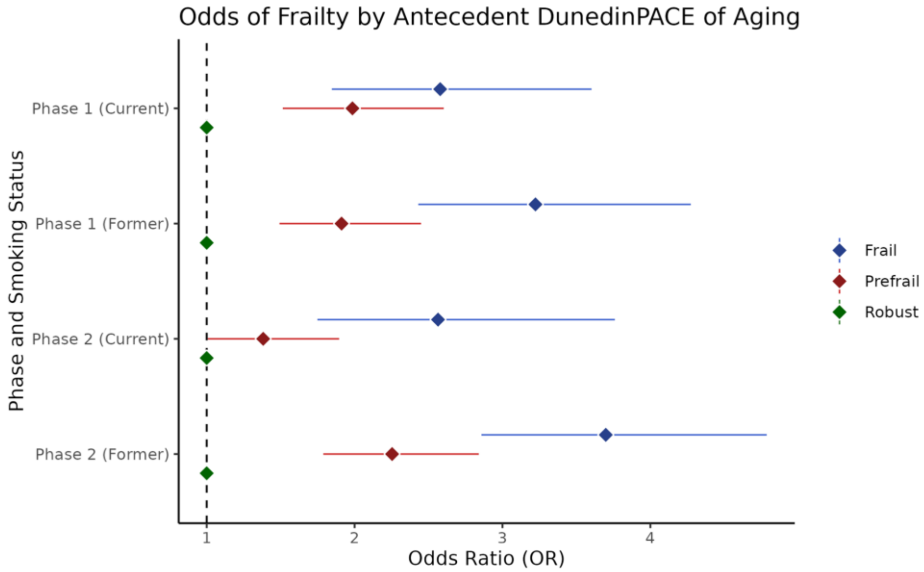

Supplementary Figure 5. Forest plot of DunedinPACE (Phase 1 and Phase 2) by frailty status (Phase 3). Odds ratios (OR) and 95% Confidence Intervals (95% CI) of DunedinPACE levels at Phase 1 (baseline) and Phase 2 (5-year follow-up) on frailty and prefrailty at Phase 3 (10-year follow-up) are shown. These are stratified by smoking status. The original units of DunedinPACE are used (one unit = one year of biological aging per year of chronological aging). Tabular format of this data is in Supplementary Table 9.

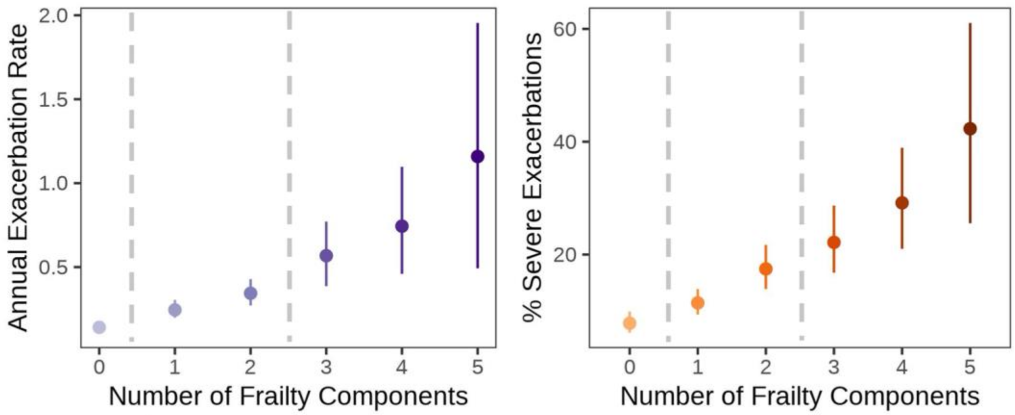

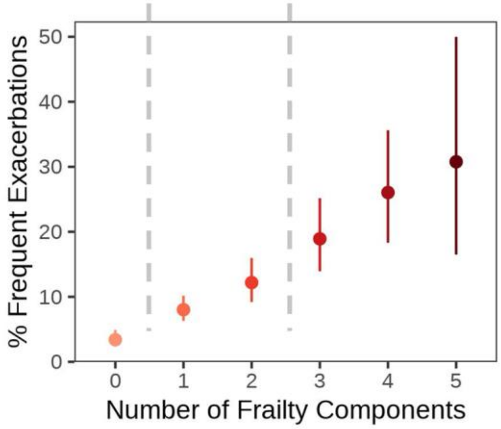

	# of Frailty Traits (n)					
	0 (793)	1 (761)	2 (361)	3 (185)	4 (96)	5 (26)
Mean Annual Exacerbation Rate (SD)	0.14 (0.50)	0.25 (0.74)	0.34 (0.78)	.57 (1.29)	.74 (1.60)	1.16 (1.98)
Severe Exacerbations (%) (95% CI)	7.8 (6.1-9.9)	11.4 (9.4-13.9)	17.5 (13.9-21.7)	22.2 (16.8-28.7)	29 (21-39)	42 (26-61)
Frequent Exacerbations (%) (95% CI)	3.4 (2.4-4.9)	8.0 (6.3-10.2)	12.2 (9.2-16.0)	18.9 (13.9-25.1)	26 (18-36)	31 (17-50)

Supplementary Figure 6. Respiratory exacerbations by number of frailty components. Top left: unadjusted annual exacerbation rate and 95% bootstrap confidence intervals among participants with *n* frailty components. Top right: % of subjects with severe exacerbations with 95% Wilson confidence intervals. Bottom left: % of subjects with frequent exacerbations with 95% Wilson confidence intervals. Dashed lines indicate the cutoffs between frailty categories (robust: 0, prefrail: 1–2, frail: 3–5). Bottom right: source data for figures.

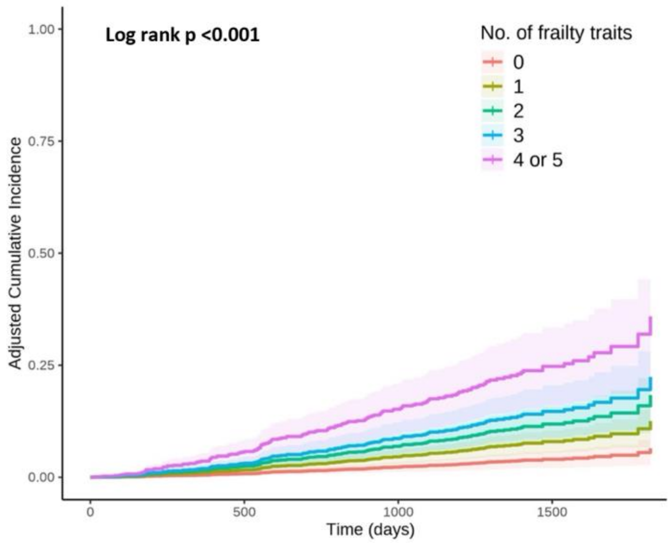

#Frailty components	AHR (95% CI)	*p*
0	-	-
1	2.1 (1.2-3.6)	**0.012**
2	3.2 (1.8-5.7)	**<0.001**
3	4.1 (2.1-7.8)	**<0.001**
4 or 5	7.6 (4.0-14.3)	**<0.001**

#Frailty components	***n* at risk**			
	Day 0	Day 500	Day 1000	Day 1500
0	865	676	521	155
1	843	607	428	132
2	420	291	200	72
3	218	140	91	23
4 or 5	147	80	56	15

Supplementary Figure 7. Mortality by number of frailty components. Adjusted all-cause mortality risk curve by the number of frailty components present. Models adjusted for age, sex, body mass index, smoking pack-years, FEV1 % predicted, diabetes, and heart disease (presence of any of: coronary artery disease, myocardial infarction, angina, angioplasty, coronary artery bypass graft surgery, or congestive heart failure). Individuals with 4 and 5 frailty components are plotted together due to small size of individuals with 5 components. Risk table is displayed beneath the cumulative incidence curve. Cox adjusted Hazard Ratios (AHR), 95% confidence intervals (95% CI), and *P*-values are displayed to the right (comparator group is robust individuals with 0 frailty traits).
