# Supplementary material for: Frailty associates with respiratory exacerbations and mortality in the COPDGene cohort
Source: Aging (Albany NY). 2025 Jul 3;17(7):1590–623. doi: 10.18632/aging.206275 (PMC12339021; doi:10.18632/aging.206275)
Supplement: Supplementary Tables [file aging-17-206275-s003.pdf]

SUPPLEMENTARY TABLES

Supplementary Table 1. Expanded participant characteristics.

Characteristic	*N*	Robust	Prefrail	Frail	*p*[a]
n (%)	2,665	912 (34%)	1,352 (51%)	401 (15%)	
Age	2,665	68.6 (7.4)	69.7 (8.1)	71.5 (9.2)	**<0.001**
Sex	2,665				0.39
Male		450 (49.3%)	640 (47.3%)	204 (50.9%)	
Female		462 (50.7%)	712 (52.7%)	197 (49.1%)	
Race	2,665				**<0.001**
Non-Hispanic White		742 (81.4%)	933 (69.0%)	270 (67.3%)	
African American		170 (18.6%)	419 (31.0%)	131 (32.7%)	
Married or Partnered	2,660	476 (52.4%)	565 (41.8%)	145 (36.3%)	**<0.001**
BMI	2,665	28.6 (5.5)	28.5 (6.1)	30.1 (7.6)	**0.002**
Current Smoking	2,663	239 (26.2%)	462 (34.2%)	138 (34.4%)	**<0.001**
Smoking Pack-Years	2,663	39.3 (20.3)	43.0 (22.4)	52.7 (26.6)	**<0.001**
GOLD grade	2,646				**<0.001**
Normal Spirometry		473 (52.3%)	589 (43.7%)	108 (27.4%)	
1		115 (12.7%)	154 (11.4%)	27 (6.9%)	
2		160 (17.7%)	293 (21.8%)	68 (17.3%)	
3		56 (6.2%)	104 (7.7%)	85 (21.6%)	
4		9 (1.0%)	32 (2.4%)	52 (13.7%)	
Total GOLD 2–4 (Moderate-Severe COPD)		225 (24.9%)	429 (31.8%)	205 (52.0%)	**<0.001**
PRISm		92 (10.2%)	175 (13.0%)	54 (13.7%)	0.08
BADL Assistance	2,664	3 (0.3%)	24 (1.8%)	34 (8.5%)	**<0.001**
IADL Assistance	2,665	6 (0.7%)	55 (4.0%)	102 (25.4%)	**<0.001**
Probable Cognitive Impairment by Mini-Cog	2,654	144 (15.9%)	325 (24.1%)	124 (31.1%)	**<0.001**
Comorbidity Count	2,665	1.1 (1.1)	1.4 (1.2)	2.0 (1.4)	**<0.001**
Diabetes		113 (12.4%)	289 (21.4%)	149 (37.2%)	**<0.001**
Coronary Artery Disease		133 (14.6%)	247 (18.3%)	118 (29.4%)	**<0.001**
Congestive Heart Failure		21 (2.3%)	54 (4.0%)	40 (10.0%)	**<0.001**
Cerebrovascular Disease		45 (4.9%)	121 (8.9%)	57 (14.2%)	**<0.001**
Kidney Disease		24 (2.6%)	61 (4.5%)	36 (9.0%)	**<0.001**
Liver Disease		36 (3.9%)	82 (6.1%)	31 (7.7%)	**0.013**
Cancer		171 (18.8%)	265 (19.6%)	91 (22.7%)	0.25
Osteoarthritis		307 (33.7%)	535 (39.6%)	204 (50.9%)	**<0.001**
Osteoporosis		132 (14.5%)	202 (14.9%)	64 (16.0%)	0.78

Total *N* = 2665. *N* with data available for each characteristic shown. Continuous variables reported as mean (standard deviation). Categorical variables reported as *n* (%). Abbreviations: BMI: body mass index (kg/m^2); GOLD: Global Initiative for Chronic Obstructive Lung Disease; COPD: Chronic Obstructive Pulmonary Disease; PRISm: preserved ratio impaired spirometry; BADL Assistance: required assistance with basic activities of daily living in the past 7 days; IADL: required assistance with independent activities of daily living in the past 7 days. Probable cognitive impairment: score of 3 or lower on the Mini-Cog screening tool. Coronary artery disease (CAD) defined as reporting any of the following: CAD, myocardial infarction, angina, angioplasty, or coronary artery bypass graft surgery. Cerebrovascular disease defined as reporting a history of stroke or transient ischemic attack (TIA). Comorbidity count is the sum of the following reported comorbidities: diabetes, coronary artery disease, congestive heart failure, cerebrovascular disease, kidney disease, liver disease, cancer (excluding non-melanoma skin cancer), osteoarthritis, and osteoporosis. [a]*p*-values for continuous variables are calculated by the Kruskal-Wallis rank sum test; for categorical variables by Pearson's chi-squared test.

Supplementary Table 2. Characteristics of never-smoker control group.

Characteristic	Never smoked (control population) (*n* = 249)	Smoking history (study population) (*N* = 2,665)	p^a
Age	66.7 (10.0)	69.6 (8.1)	**<0.001**
Sex			0.34
Male	113 (45.4%)	1,294 (48.6%)	
Female	136 (54.6%)	1,371 (51.4%)	
Race			**<0.001**
Non-Hispanic White	215 (86.3%)	1,945 (73.0%)	
African American	34 (13.7%)	720 (27.0%)	
BMI	27.7 (4.8)	28.8 (6.1)	**0.02**
FEV1 (% predicted)	102.3 (14.9)	80.2 (24.7)	**<0.001**
Comorbidity Count	0.7 (0.9)	1.4 (1.2)	**<0.001**
Diabetes	21 (8.4%)	551 (20.7%)	**<0.001**
Coronary Artery Disease	10 (4.0%)	498 (18.7%)	**<0.001**
Congestive Heart Failure	1 (0.4%)	115 (4.3%)	**0.003**
Cerebrovascular Disease	6 (2.4%)	223 (8.4%)	**<0.001**
Kidney Disease	2 (0.8%)	121 (4.5%)	**0.005**
Liver Disease	2 (0.8%)	149 (5.6%)	**0.001**
Cancer	43 (17.3%)	527 (19.8%)	0.34
Osteoarthritis	69 (27.7%)	1,046 (39.2%)	**<0.001**
Osteoporosis	28 (11.2%)	398 (14.9%)	0.12
Frailty Category			**<0.001**
Robust	161 (64.7%)	912 (34.2%)	
Prefrail	84 (33.7%)	1,352 (50.7%)	
Frail	4 (1.6%)	401 (15.0%)	

Characteristics of the never-smoker group (evaluated in a post hoc analysis) compared with the study population. Continuous variables reported as mean (standard deviation). Categorical variables reported as *n* (%). [a]*p*-values for continuous variables are calculated by the Wilcoxon rank sum test; for categorical variables by Pearson's chi-squared test.

Supplementary Table 3. Prevalence of frailty components.

	Prevalence (%)																			
	All subjects				Normal Spirometry				GOLD 1				GOLD 2–4				PRISm			
	All	Rob	Pre	Frail	All	Rob	Pre	Frail	All	Rob	Pre	Frail	All	Rob	Pre	Frail	All	Rob	Pre	Frail
N	2665	912	1352	401	1170	473	589	108	296	115	154	27	859	225	429	205	321	92	175	54
Fatigue	19.5	–	19.9	62.8	15.6	–	18.3	69.4	17.2	–	20.8	70	23.5	–	19.1	58.5	24.3	–	25.1	63
Slowness	23.0	–	21.4	80.5	13.8	–	16.1	62.0	15.9	–	16.2	81	34.5	–	27.0	87.8	30.8	–	29.1	89
Weakness	29.7	–	37.6	70.8	25.6	–	36.7	77.8	25.0	–	36.4	67	34.0	–	36.1	66.8	37.4	–	46.3	72
Low Activity	15.1	–	9.7	67.6	8.0	–	6.6	50.9	8.8	–	6.5	59	25.5	–	14.2	77.1	18.4	–	12.0	70
Shrinking	33.1	–	45.5	66.6	33.4	–	51.6	80.6	32.8	–	52.6	59	37.3	–	43.8	64.4	21.5	–	23.4	52

Prevalence of the five frailty components among prefrail and frail participants. For robust participants, the prevalence is 0% by definition. Prevalence is reported in the entire study cohort and by spirometric subgroup. *N* with frailty measurements but missing spirometry = 19. Rob = robust, Pre = prefrail. GOLD = Global Initiative for Chronic Obstructive Lung Disease. PRISm = Preserved Ratio Impaired Spirometry.

Supplementary Table 4. Characteristics of participants with missing follow-up data.

	Missing mortality data			Missing exacerbation data		
**Characteristic**	**Analyzed (*n* = 2512)**	**Missing (*n* = 153)**	***p*[a]**	**Analyzed (*n* = 2222)**	**Missing (*n* = 443)**	***p*[a]**
Age	69.7 (8.2)	68.0 (7.2)	**0.009**	69.8 (8.1)	68.7 (8.1)	**0.003**
Sex			0.91			**0.03**
Male	1,219 (48.5%)	75 (49.0%)		1,058 (47.6%)	236 (53.3%)	
Female	1,293 (51.5%)	78 (51.0%)		1,164 (52.4%)	207 (46.7%)	
Race			**<0.001**			**<0.001**
Non-Hispanic White	1,869 (74.4%)	76 (49.7%)		1,698 (76.4%)	247 (55.8%)	
African American	643 (25.6%)	77 (50.3%)		524 (23.6%)	196 (44.2%)	
BMI	28.8 (6.1)	28.5 (6.2)	0.52	28.9 (6.2)	28.6 (6.0)	0.54
Current Smoker	771 (30.7%)	68 (44.4%)	**<0.001**	662 (29.8%)	177 (40.0%)	**<0.001**
Smoking Pack-Years	43.3 (22.9)	42.5 (22.6)	0.64	43.1 (22.7)	43.9 (23.7)	0.61
GOLD grade			0.61			0.81
Normal Spirometry	1,106 (44.3%)	64 (42.4%)		976 (44.2%)	194 (44.4%)	
1	280 (11.2%)	16 (10.6%)		252 (11.4%)	44 (10.1%)	
2	489 (19.6%)	32 (21.2%)		435 (19.7%)	86 (19.7%)	
3	233 (9.3%)	12 (7.9%)		200 (9.1%)	45 (10.3%)	
4	90 (3.6%)	3 (2.0%)		81 (3.7%)	12 (2.7%)	
PRISm	297 (11.9%)	24 (15.9%)		265 (12.0%)	56 (12.8%)	
Comorbidity Count	1.4 (1.2)	1.3 (1.2)	0.79	1.4 (1.2)	1.4 (1.3)	0.55
Diabetes	515 (20.5%)	36 (23.5%)	0.37	439 (19.8%)	112 (25.3%)	**0.009**
Coronary Artery Disease	474 (18.9%)	24 (15.7%)	0.33	422 (19.0%)	76 (17.2%)	0.37
Congestive Heart Failure	108 (4.3%)	7 (4.6%)	0.87	90 (4.1%)	25 (5.6%)	0.13
Cerebrovascular Disease	204 (8.1%)	19 (12.4%)	0.06	179 (8.1%)	44 (9.9%)	0.19
Kidney Disease	109 (4.3%)	12 (7.8%)	**0.04**	92 (4.1%)	29 (6.5%)	**0.03**
Liver Disease	139 (5.5%)	10 (6.5%)	0.60	107 (4.8%)	42 (9.5%)	**<0.001**
Cancer		28 (18.3%)		440 (19.8%)	87 (19.6%)	0.94
Osteoarthritis	993 (39.5%)	53 (34.6%)	0.23	893 (40.2%)	153 (34.5%)	**0.03**
Osteoporosis	381 (15.2%)	17 (11.1%)	0.17	345 (15.5%)	53 (12.0%)	0.055
Frailty Category			**0.04**			**<0.001**
Robust	872 (34.7%)	40 (26.1%)		793 (35.7%)	119 (26.9%)	
Prefrail	1,270 (50.6%)	82 (53.6%)		1,122 (50.5%)	230 (51.9%)	
Frail	370 (14.7%)	31 (20.3%)		307 (13.8%)	94 (21.2%)	

Characteristics of individuals with missing longitudinal follow-up data (exacerbations and mortality data) compared with those with follow-up data. Continuous variables reported as mean (standard deviation). Categorical variables reported as *n* (%). Abbreviations: GOLD: Global Initiative for Chronic Obstructive Lung Disease; COPD: Chronic Obstructive Pulmonary Disease; PRISm: Preserved Ratio Impaired Spirometry. [a]*p*-values for continuous variables are calculated by the Wilcoxon rank sum test; for categorical variables by Pearson's chi-squared test.

Supplementary Table 5. Odds of respiratory exacerbations by frailty and spirometric category.

(A) Adjusted models

	All subjects					Normal Spirometry					GOLD 2–4				
	**Robust (*n* = 787)**	**Prefrail (*n* = 1119)**		**Frail (*n* = 303)**		**Robust (*n* = 407)**	**Prefrail (*n* = 489)**		**Frail (*n* = 80)**		**Robust (*n* = 198)**	**Prefrail (*n* = 358)**		**Frail (*n* = 160)**	
	**OR/IRR**	**OR/IRR**	***p***	**OR/IRR**	***p***	**OR/IRR**	**OR/IRR**	***p***	**OR/IRR**	***p***	**OR/IRR**	**OR/IRR**	***p***	**OR/IRR**	***p***
Exacerbation Rate	1	**1.8 (1.4–2.3)**	**<0.001**	**3.4 (2.4–4.8)**	**<0.001**	1	**2.0 (1.2–3.2)**	**0.006**	**4.2 (1.8–9.6)**	**<0.001**	**1**	**2.6 (1.8–3.7)**	**<0.001**	**3.9 (2.5–6.2)**	**<0.001**
Severe Exacerbations	1	**1.6 (1.1–2.2)**	**0.005**	**2.8 (1.8–4.2)**	**<0.001**	1	1.6 (0.9–3.0)	0.10	**3.4 (1.5–7.7)**	**0.004**	**1**	**1.9 (1.1–3.1)**	**0.02**	**2.7 (1.5–5.1)**	**0.002**
Frequent Exacerbations	1	**2.6 (1.7–4.1)**	**<0.001**	**5.5 (3.2–9.3)**	**<0.001**	1	**3.2 (1.4–7.1)**	**0.004**	**9.1 (3.0–27.9)**	**<0.001**	**1**	**4.3 (2.0–9.4)**	**<0.001**	**9.8 (4.1–23.5)**	**<0.001**

	GOLD 1					PRISm				
	**Robust (*n* = 104)**	**Prefrail (*n* = 127)**		**Frail (*n* = 21)**		**Robust (*n* = 78)**	**Prefrail (*n* = 145)**		**Frail (*n* = 42)**	
	**OR/IRR**	**OR/IRR**	***p***	**OR/IRR**	***p***	**OR/IRR**	**OR/IRR**	***p***	**OR/IRR**	***p***
Exacerbation Rate	1	1.2 (0.6–2.4)	0.68	2.6 (0.7–9.1)	0.15	1	1.1 (0.5–2.4)	0.90	2.5 (.99–6.1)	0.051
Severe Exacerbations	1	1.1 (0.5–2.5)	0.83	1.9 (0.6–6.7)	0.31	1	1.3 (0.5–3.7)	0.56	**3.2 (1.01–10.3)**	**0.047**
Frequent Exacerbations	1	1.5 (0.5–4.6)	0.47	–	–	1	0.8 (0.3–2.7)	0.78	1.9 (0.5–7.4)	0.38

(B) Unadjusted models

	All subjects					Normal Spirometry					GOLD 2–4				
	**Robust**	**Prefrail**		**Frail**		**Robust**	**Prefrail**		**Frail**		**Robust**	**Prefrail**		**Frail**	
	**OR/IRR**	**OR/IRR**	***p***	**OR/IRR**	***p***	**OR/IRR**	**OR/IRR**	***p***	**OR/IRR**	***p***	**OR/IRR**	**OR/IRR**	***p***	**OR/IRR**	***p***
Exacerbation Rate	1	**2.1 (1.6–2.7)**	**<0.001**	**5 (3.6–6.9)**	**<0.001**	1	**2.1 (1.3–3.3)**	**0.003**	**4.3 (1.9–9.7)**	**<0.001**	1	**2.5 (1.7–3.6)**	**<0.001**	**4.8 (3.2–7.2)**	**<0.001**
Severe Exacerbations	1	**1.8 (1.3–2.5)**	**<0.001**	**4.2 (2.9–6)**	**<0.001**	1	1.7 (1–3.1)	0.07	**3.8 (1.8–8.3)**	**<0.001**	1	**2.0 (1.2–3.3)**	**0.005**	**3.4 (2.0–5.8)**	**<0.001**
Frequent Exacerbations	1	**2.9 (1.9–4.5)**	**<0.001**	**8.1 (5.1–12.9)**	**<0.001**	1	**3.3 (1.5–7.2)**	**0.003**	**6.3 (2.4–16.9)**	**<0.001**	1	**4.5 (2.1–9.6)**	**<0.001**	**11.1 (5.1–24.3)**	**<0.001**

	GOLD 1					PRISm				
	**Robust**	**Prefrail**		**Frail**		**Robust**	**Prefrail**		**Frail**	
	**OR/IRR**	**OR/IRR**	***p***	**OR/IRR**	***p***	**OR/IRR**	**OR/IRR**	***p***	**OR/IRR**	***p***
Exacerbation Rate	1	1.1 (0.5–2.2)	0.87	3.0 (0.9–10.4)	0.08	1	1.2 (0.5–2.6)	0.73	2.5 (1.0–6.1)	0.06
Severe Exacerbations	1	1.0 (0.5–2.2)	0.98	2.2 (0.7–7.0)	0.19	1	1.6 (0.6–4.2)	0.35	**3.3 (1.1–10)**	**0.04**
Frequent Exacerbations	1	1.2 (0.4–3.6)	0.69	-	-	1	1 (0.3–3.0)	0.95	2.0 (0.5–7.2)	0.31

Odds ratio of frailty (vs robustness) and prefrailty (vs robustness) on exacerbations for all subjects and for subgroups of participants by spirometric category. Abbreviations: OR: odds ratio; IRR: incident rate ratio; GOLD: Global Initiative for Chronic Obstructive Lung Disease; PRISm: Preserved Ratio Impaired Spirometry. N is # of individuals within each category who had over 180 days of follow-up exacerbation data *and* spirometry collected. Reported as OR/IRR (95% confidence interval). (**A**) Models adjusted for age, sex, current smoking status, and forced expiratory volume in 1 second (FEV1) %predicted. OR for frailty not calculated for frequent exacerbations in GOLD 1 subgroup due to low cell count (<5). (**B**) Results from unadjusted model.

Supplementary Table 6. Crude and adjusted all-cause mortality.

(A) Crude event counts and log-rank *p*-values for all-cause mortality by spirometric category

	All subjects				Normal Spirometry				GOLD 1				GOLD 2–4				PRISm			
	Rob	Pre	Frail	p^a	Rob	Pre	Frail	*p*	Rob	Pre	Frail	*p*	Rob	Pre	Frail	*p*	Rob	Pre	Frail	*p*
# Events	18	70	58	**<0.001**	4	20	8	**<0.001**	2	8	3	**0.049**	9	37	42	**<0.001**	3	5	4	0.2
n	872	1270	370		449	556	101		111	143	26		215	406	191		90	160	47	

(B) Unadjusted and adjusted hazard ratios of frailty and prefrailty for all-cause mortality

	All subjects				GOLD 2–4				GOLD 0			
	Unadjusted		Adjusted		Unadjusted		Adjusted		Unadjusted		Adjusted	
	HR (95% CI)	p^b	AHR (95% CI)	*p*	HR (95% CI)	*p*	AHR (95% CI)	*p*	HR (95% CI)	*p*	AHR (95% CI)	*p*
Prefrailty	**3.0 (1.8–5.1)**	**<0.001**	**2.5 (1.5–4.2)**	**<0.001**	**2.5 (1.2–5.2)**	**0.014**	**2.1 (1.02–4.4)**	**0.045**	**5.0 (1.7–14.5)**	**0.003**	**4.2 (1.4–12.6)**	**0.01**
Frailty	**9.9 (5.8–16.8)**	**<0.001**	**4.5 (2.4–8.5)**	**<0.001**	**7.1 (3.5–14.7)**	**<0.001**	**4.0 (1.7–9.3)**	**0.001**	**15.4 (4.6–51.6)**	**<0.001**	**7.9 (1.9–32.5)**	**0.004**

(**A**) Crude event counts and log-rank *p*-values for all-cause mortality by spirometric category. Abbreviations: Rob: robust; Pre: prefrail; GOLD: Global Initiative for Chronic Obstructive Lung Disease. PRISm: Preserved Ratio Impaired Spirometry. [a]log-rank *p*-value for frailty category (frail, prefrail, or robust) vs all-cause mortality. (**B**) Unadjusted and adjusted hazard ratios of frailty and prefrailty for all-cause mortality. Unadjusted Hazard Ratios (HR), adjusted Hazard Ratios (AHR), 95% confidence intervals (95% CI) and [b]*p*-values from Cox models are reported overall and for the largest spirometric subgroups. For both frailty and prefrailty, robust groups were the comparator. Adjusted Cox models adjusted for age, sex, body mass index, smoking pack-years, forced expiratory volume in one second (FEV1) % predicted, diabetes, and heart disease (presence of any of: coronary artery disease, myocardial infarction, angina, angioplasty, coronary artery bypass graft surgery, or congestive heart failure).

Supplementary Table 7. Characteristics of participants with missing DNA methylation data.

Characteristic	Missing DNA Methylation data (Either Phase)			Missing DNA Methylation data (Phase 1)			Missing DNA Methylation data (Phase 2)		
	Analyzed (*n* = 2104)	Missing (*n* = 561)	p^a	Present (*n* = 2247)	Missing (*n* = 418)	p^a	Present (*n* = 2376)	Missing (*n* = 289)	p^a
Age	69.4 (8.1)	70.3 (8.1)	**0.02**	69.4 (8.1)	70.6 (8.0)	**0.004**	69.6 (8.1)	69.3 (8.1)	0.51
Sex			0.61			0.83			0.93
Male	1,027 (48.8%)	267 (47.6%)		1,089 (48.5%)	205 (49.0%)		1,153 (48.5%)	141 (48.8%)	
Female	1,077 (51.2%)	294 (52.4%)		1,158 (51.5%)	213 (51.0%)		1,223 (51.5%)	148 (51.2%)	
Race			**0.01**			**0.02**			**<0.001**
Non-Hispanic White	1,559 (74.1%)	386 (68.8%)		1,659 (73.8%)	286 (68.4%)		1,761 (74.1%)	184 (63.7%)	
African American	545 (25.9%)	175 (31.2%)		588 (26.2%)	132 (31.6%)		615 (25.9%)	105 (36.3%)	
BMI	28.9 (6.2)	28.6 (6.1)	0.24	28.9 (6.2)	28.5 (5.7)	0.32	28.8 (6.1)	28.6 (6.4)	0.21
Current Smoker	672 (32.0%)	167 (29.8%)	0.32	719 (32.0%)	120 (28.7%)	0.18	745 (31.4%)	94 (32.5%)	0.69
Smoking Pack-Years	43.0 (22.7)	44.0 (23.5)	0.52	43.0 (22.8)	44.2 (23.1)	0.38	43.0 (22.6)	45.2 (24.6)	0.27
GOLD grade			**0.03**			**0.006**			0.20
Normal Spirometry	907 (43.4%)	263 (47.2%)		973 (43.6%)	197 (47.5%)		1,047 (44.4%)	123 (42.9%)	
1	236 (11.3%)	60 (10.8%)		250 (11.2%)	46 (11.1%)		268 (11.4%)	28 (9.8%)	
2	429 (20.5%)	92 (16.5%)		459 (20.6%)	62 (14.9%)		464 (19.7%)	57 (19.9%)	
3	188 (9.0%)	57 (10.2%)		205 (9.2%)	40 (9.6%)		214 (9.1%)	31 (10.8%)	
4	65 (3.1%)	28 (5.0%)		68 (3.0%)	25 (6.0%)		76 (3.2%)	17 (5.9%)	
PRISm	264 (12.6%)	57 (10.2%)		276 (12.4%)	45 (10.8%)		290 (12.3%)	31 (10.8%)	
Comorbidity Count	1.3(1.2)	1.5(1.3)	**0.05**	1.3(1.2)	1.4(1.3)	0.21	1.4(1.2)	1.4(1.3)	0.27
Diabetes	430 (20.4%)	121 (21.6%)	0.56	462 (20.6%)	89 (21.3%)	0.74	484 (20.4%)	67 (23.2%)	0.27
Coronary Artery Disease	378 (18.0%)	120 (21.4%)	0.06	406 (18.1%)	92 (22.0%)	0.06	433 (18.2%)	65 (22.5%)	0.08
Congestive Heart Failure	89 (4.2%)	26 (4.6%)	0.68	97 (4.3%)	18 (4.3%)	0.99	99 (4.2%)	16 (5.5%)	0.28

Cerebrovascular Disease	164 (7.8%)	59 (10.5%)	**0.04**	179 (8.0%)	44 (10.5%)	0.08	190 (8.0%)	33 (11.4%)	**0.05**
Kidney Disease	95 (4.5%)	26 (4.6%)	0.90	104 (4.6%)	17 (4.1%)	0.61	108 (4.5%)	13 (4.5%)	0.97
Liver Disease	102 (4.8%)	47 (8.4%)	**0.001**	111 (4.9%)	38 (9.1%)	**<0.001**	125 (5.3%)	24 (8.3%)	**0.03**
Cancer	405 (19.2%)	122 (21.7%)	0.19	444 (19.8%)	83 (19.9%)	0.96	465 (19.6%)	62 (21.5%)	0.45
Osteoarthritis	830 (39.4%)	216 (38.5%)	0.68	894 (39.8%)	152 (36.4%)	0.19	940 (39.6%)	106 (36.7%)	0.34
Osteoporosis	313 (14.9%)	85 (15.2%)	0.87	329 (14.6%)	69 (16.5%)	0.33	365 (15.4%)	33 (11.4%)	0.08
Frailty Category			0.10			0.37			**0.004**
Robust	738 (35.1%)	174 (31.0%)		781 (34.8%)	131 (31.3%)		835 (35.1%)	77 (26.6%)	
Prefrail	1,062 (50.5%)	290 (51.7%)		1,128 (50.2%)	224 (53.6%)		1,198 (50.4%)	154 (53.3%)	
Frail	304 (14.4%)	97 (17.3%)		338 (15.0%)	63 (15.1%)		343 (14.4%)	58 (20.1%)	

Characteristics of individuals with missing methylation data at baseline (Phase 1) or 5-year follow-up (Phase 2) compared with those with methylation data. Continuous variables reported as mean (standard deviation). Categorical variables reported as *n* (%). Abbreviations: GOLD: Global Initiative for Chronic Obstructive Lung Disease; COPD: Chronic Obstructive Pulmonary Disease; PRISm: Preserved Ratio Impaired Spirometry. [a]*p*-values for continuous variables are calculated by the Wilcoxon rank sum test; for categorical variables by Pearson's chi-squared test. While complete case analysis was performed (only individuals with methylation data at both Phase 1 and Phase 2 were included in analysis – left hand columns), participant characteristics based on methylation missingness by Phase are also reported for completeness (right hand columns).

Supplementary Table 8. DunedinPACE of Epigenetic Aging (Phases 1 and 2) by frailty status at phase 3 (stratified by smoking status).

		Frailty Status at Phase 3							
		**Robust**	**Prefrail**	**Frail**	***p***	**Robust**	**Prefrail**	**Frail**	***p***
**Phase 1**	**Smoking Status (at Phase 1)**	Former (Phase 1)				Current (Phase 1)			
	n	431	576	159		307	486	145	
	**Mean Age (SD)**	60 (7)	62 (8)	66 (8)		54 (7)	55 (7)	55 (7)	
	**DunedinPACE**	1.01 (0.12)	1.05 (0.12)	1.10 (0.11)	**<0.001**	1.10 (0.12)	1.14 (0.12)	1.17 (0.13)	**<0.001**
**Phase 2**	**Smoking Status (at Phase 2)**	Former (Phase 2)				Current (Phase 2)			
	n	497	660	187		241	402	117	
	**Mean Age (SD)**	65 (7)	67 (8)	70 (9)		60 (7)	60 (7)	61 (7)	
	**DunedinPACE**	1.02 (0.11)	1.07 (0.12)	1.12 (0.12)	**<0.001**	1.12 (0.11)	1.14 (0.12)	1.18 (0.13)	**<0.001**

The DunedinPACE of epigenetic aging (units represent biological years aged per chronological year aged) as measured by methylation data collected at baseline (Phase 1) and at 5-year follow up (Phase 2) stratified by smoking status at time of blood draw. DunedinPACE is reported as mean (standard deviation). This is reported by frailty status at 10-year (Phase 3) follow-up. *p*-values were calculated by Kruskal-Wallis rank sum test across the frailty categories. For reference, mean age (standard deviation) at each Phase is also reported across smoking status category and frailty category.

Supplementary Table 9. Odds of frailty at phase 3 by DunedinPACE of Aging (Phases 1 and 2) (stratified by smoking status).

		Prefrailty		Frailty	
**Phase**	**Smoking Status**	**OR (95% CI)**	***p***	**OR (95% CI)**	***p***
1	Current	1.99 (1.51–2.60)	**<0.001**	2.58 (1.85–3.60)	**<0.001**
	Former	1.91 (1.49–2.45)	**<0.001**	3.22 (2.43–4.27)	**<0.001**
	Overall	1.90 (1.60–2.25)	**<0.001**	2.78 (2.26–3.40)	**<0.001**
2	Current	1.38 (1.01–1.90)	**0.045**	2.56 (1.75–3.76)	**<0.001**
	Former	2.25 (1.79–2.84)	**<0.001**	3.70 (2.86–4.79)	**<0.001**
	Overall	1.91 (1.60–2.28)	**<0.001**	3.05 (2.49–3.73)	**<0.001**

OR (odds ratio) and 95% CI (95% Confidence Interval) of blood DunedinPACE (epigenetic pace of aging – one unit represents one biological year aged per chronological year aged) on the outcome of subsequent prefrailty and frailty (compared to robustness) is calculated. The odds of DunedinPACE from Phase 1 (baseline) blood draw and from Phase 2 (5-year) blood draw on Phase 3 (10-year) frailty and prefrailty are reported. Results are stratified by smoking status at the time of blood draw.

Supplementary Table 10. Odds of respiratory exacerbations by number of frailty components.

**Outcome**	**# Frailty components**	**Unadjusted**		**Adjusted**	
		**OR/IRR (95% CI)**	***p***	**OR/IRR (95% CI)**	***p***
Exacerbation Rate	0	–	–	–	–
	1	**1.8 (1.4–2.4)**	**<0.001**	**1.7 (1.3–2.2)**	**<0.001**
	2	**2.6 (1.9–3.6)**	**<0.001**	**2.0 (1.5–2.8)**	**<0.001**
	3	**4.2 (2.8–6.3)**	**<0.001**	**3.2 (2.1–4.7)**	**<0.001**
	4	**5.5 (3.2–9.3)**	**<0.001**	**3.2 (1.9–5.3)**	**<0.001**
	5	**8.9 (3.5–22.5)**	**<0.001**	**5.3 (2.1–12.9)**	**<0.001**
Severe Exacerbations	0	–	–	–	–
	1	**1.5 (1.1–2.1)**	**0.02**	**1.5 (1.0–2.1)**	**0.04**
	2	**2.5 (1.7–3.6)**	**<0.001**	**2 (1.3–2.9)**	**<0.001**
	3	**3.4 (2.2–5.2)**	**<0.001**	**2.3 (1.5–3.7)**	**<0.001**
	4	**4.9 (2.9–8.1)**	**<0.001**	**2.5 (1.4–4.3)**	**0.002**
	5	**8.6 (3.8–19.6)**	**<0.001**	**4.3 (1.7–10.4)**	**0.001**
Frequent Exacerbations	0	–	–	–	–
	1	**2.5 (1.6–3.9)**	**<0.001**	**2.4 (1.5–3.9)**	**<0.001**
	2	**3.9 (2.4–6.5)**	**<0.001**	**3.2 (1.9–5.3)**	**<0.001**
	3	**6.6 (3.9–11.3)**	**<0.001**	**4.9 (2.8–8.6)**	**<0.001**
	4	**10.0 (5.5–18.1)**	**<0.001**	**5.2 (2.7–10.0)**	**<0.001**
	5	**12.6 (5–31.5)**	**<0.001**	**6.6 (2.4–18.0)**	**<0.001**

Crude and adjusted odds ratios (OR) and 95% confidence interval (95% CI) of the number of frailty components (0–5, treated categorically using 0 as comparator) on odds of severe and frequent exacerbations, and crude and adjusted incident rate ratio (IRR) of the number of frailty components on exacerbation rate. Covariates in adjusted models: age, sex, smoking status, and forced expiratory volume in 1 second (FEV1) %predicted (exacerbation rate models also included an offset term for the log(follow-up time)). (See Supplementary Figure 4 for raw exacerbation counts).

Supplementary Table 11. Sex-stratified analysis of frailty category on respiratory exacerbations and mortality.

	Prefrailty				Frailty			
	Men		Women		Men		Women	
	OR/IRR/AHR	*p*	OR/IRR/AHR	*p*	OR/IRR/AHR	*p*	OR/IRR/AHR	*p*
Exacerbation Rate	1.3 (0.9–1.9)	0.24	**2.5 (1.8–3.5)**	**<0.001**	**2.3 (1.3–4.1)**	**0.003**	**4.7 (3.1–7.2)**	**<0.001**
Severe Exacerbations	1.3 (0.8–2.0)	0.32	**1.9 (1.2–3.1)**	**0.004**	**2.2 (1.2–4.1)**	**0.01**	**3.4 (1.9–6.0)**	**<0.001**
Frequent Exacerbations	**1.9 (1.0–3.3)**	**0.04**	**4.0 (2.0–8.0)**	**<0.001**	**3.6 (1.7–7.6)**	**<0.001**	**8.9 (4.1–19.6)**	**<0.001**
Mortality	**2.4 (1.3–4.6)**	**0.01**	**2.5 (1.0–6.2)**	**0.05**	**4.3 (2.0–9.2)**	**<0.001**	**5.0 (1.7–14.5)**	**0.003**

Odds ratio (OR - severe exacerbations and frequent exacerbations), incident rate ratio (IRR - exacerbation rate), and adjusted hazard ratio (AHR; mortality from Cox proportional hazard model) are reported as OR/IRR/AHR (95% confidence interval).

Supplementary Table 12. Frailty category vs. Odds of respiratory exacerbations stratified by time of Phase 3 visit relative to the Covid-19 pandemic (Sensitivity Analysis).

	Pre-Pandemic (n = 1596)					Post-Pandemic (n = 626)				
	Robust (n = 588)	Prefrail (n = 790)	Frail (n = 218)	Prefrailty OR/IRR	Frailty IRR/OR	Robust (n = 205)	Prefrail (n = 332)	Frail (n = 89)	Prefrailty OR/IRR	Frailty OR/IRR
Annual exacerbation rate	0.14 (0.45)	0.28 (0.71)	**0.64**[a] (1.15)	**1.8 (1.4–2.4)**	**3.0 (2.1–4.4)**	.14 (.61)	.28 (.86)	**.76**[a] (2.0)	1.8 (0.97–3.5)	**4.8 (1.9–11.9)**
Severe exacerbations	9.2% (54)	15.6% (123)	**29.8%**[a] (65)	**1.6 (1.1–2.2)**	**2.6 (1.7–4.2)**	3.9% (8)	8.1% (27)	**17%**[a] (15)	2.0 (0.9–4.7)	**4.5 (1.6–12.5)**
Frequent exacerbations	2.9% (17)	9.5% (75)	**22.9%**[a] (50)	**3.1 (1.8–5.4)**	**6.5 (3.4–12.3)**	4.9% (10)	9.0% (30)	**20%**[a] (18)	1.7 (0.8–3.6)	**3.6 (1.4–9.3)**

Annual exacerbation rate reported as mean (standard deviation). Frequencies reported as % (n). For exacerbation rate, the incident rate ratio (IRR) (95% CI) is reported. For severe and frequent exacerbations, the odds ratio (OR) (95% CI) is reported. Frailty and prefrailty OR/IRR are compared to robust group. [a]*p*-value < 0.05 across frailty category by Kruskal-Wallis rank sum test for exacerbation rate or Pearson's chi-squared test for severe and frequent exacerbations.
